# Donor Radii in Rare-Earth Complexes

**DOI:** 10.1021/acs.inorgchem.3c03126

**Published:** 2023-10-02

**Authors:** Charlene Harriswangler, Juan C. Frías, M. Teresa Albelda, Laura Valencia, Enrique García-España, David Esteban-Gómez, Carlos Platas-Iglesias

**Affiliations:** † Centro Interdisciplinar de Química e Bioloxía (CICA) and Departamento de Química, Facultade de Ciencias, Universidade da Coruña, A Coruña 15071, Galicia , Spain; ‡ Departamento de Ciencias Biomédicas, Universidad Cardenal Herrera-CEU, CEU Universities, 46115 Valencia, Spain; § Instituto de Ciencia Molecular (ICMol), Departamento de Química Inorgánica, Universidad de Valencia, 46980 Paterna, Spain; ∥ Departamento de Química Inorgánica, Facultad de Ciencias, Universidade de Vigo, As Lagoas, Marcosende, 36310 Pontevedra, Spain; ▽ Departamento de Química Inorgánica, Universidad de Valencia, C/Dr. Moliner 50, 46100 Burjasot, Valencia, Spain

## Abstract

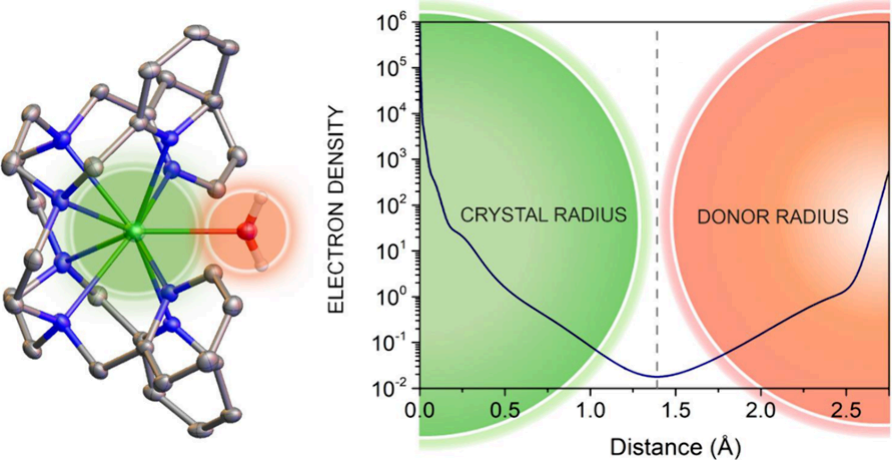

We present a set of donor radii for the rare-earth cations obtained from the analysis of structural data available in the Cambridge Structural Database (CSD). Theoretical calculations using density functional theory (DFT) and wave function approaches (NEVPT2) demonstrate that the Ln-donor distances can be broken down into contributions of the cation and the donor atom, with the minimum in electron density (ρ) that defines the position of (3,–1) critical points corresponding well with Shannon’s crystal radii (CR). Subsequent linear fits of the experimental bond distances for all rare earth cations (except *Pm*^3+^) afforded donor radii (*r*_D_) that allow for the prediction of Ln-donor distances regardless of the nature of the rare-earth cation and its oxidation state. This set of donor radii can be used to rationalize structural data and identify particularly weak or strong interactions, which has important implications in the understanding of the stability and reactivity of complexes of these metal ions. A few cases of incorrect atom assignments in X-ray structures were also identified using the derived *r*_D_ values.

## Abstract 2

Lanthanide-donor distances can be estimated by using crystal radii and donor radii, which were obtained from the simultaneous analysis of a large number of X-ray structures of lanthanide complexes.

## Introduction

**Chart 1:**
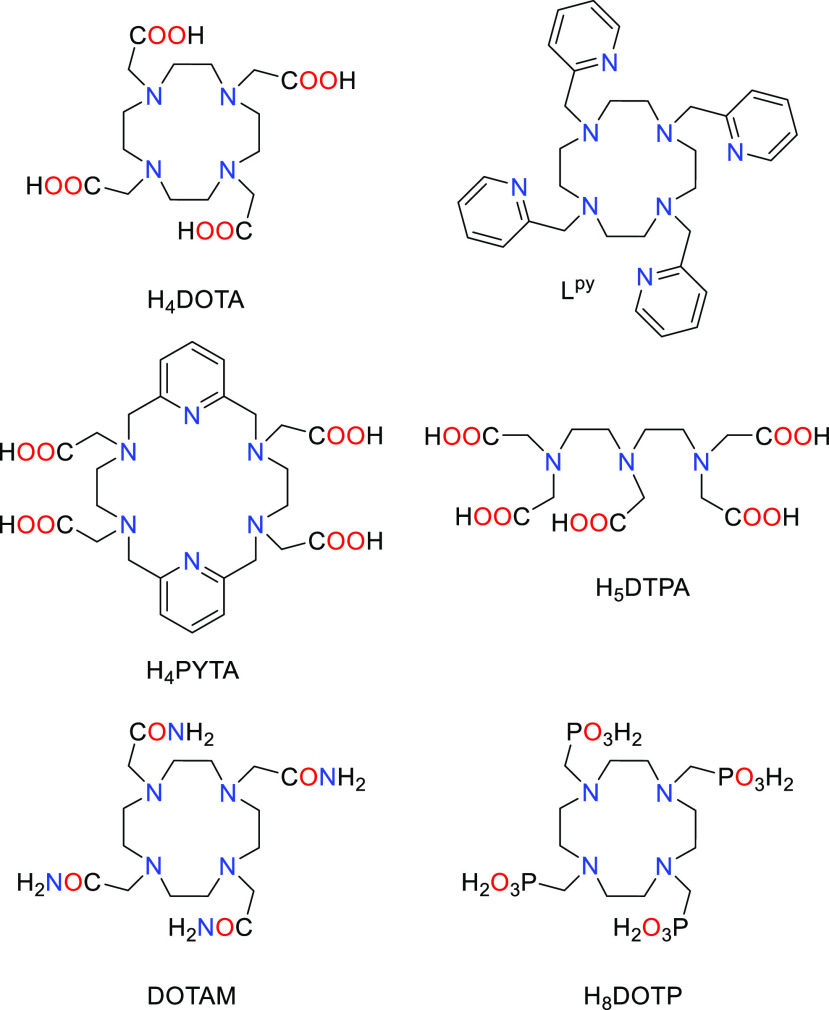
Structures of Ligands Discussed in the Present Work

The lanthanide series comprises a very coherent group of elements of the periodic table from La (*Z* = 57) to Lu (*Z* = 71), whose coordination chemistry is dominated by the trivalent oxidation state.^1^ The Ln(III) ions are characterized by their high hydration energies^2,3^ and their hard character according to Pearson’s classification.^4^ Thus, stable complexation of the Ln(III) ions is generally achieved in aqueous media with hard polyaminopolycarboxilate ligands. The high coordination numbers adopted by these ions (often 8–9)^5,6^ make octadentate ligands such as H_5_DTPA^7^ and H_4_DOTA,^8^ particularly useful for stable complexation in aqueous media (Chart 1).^9,10^ Complexes of these ligands and closely related derivatives are currently used for different biomedical and bioanalytical applications, including the clinical use of Gd(III) complexes in magnetic resonance imaging^11^ and a DOTA derivative in radiotherapy using the β-emitter ^177^Lu-radionuclide.^12^ Some Ln(III) ions are also widely used as luminescent tags for bioanalytical purposes, in particular Eu(III) and Tb(III) complexes, which emit in the visible region of the spectrum.^13,14^ Luminescence upconversion has also been recently achieved in molecular Ln(III) complexes.^15,16^ Furthermore, the magnetic properties of Ln(III) molecular complexes are currently receiving a great deal of attention due to their behavior as single-molecule magnets.^17,18^

The applications described above triggered intense research efforts devoted to the preparation of a wide variety of H_5_DTPA and H_4_DOTA lanthanide derivatives for different purposes. The Cambridge Structural Database (CSD) currently contains around 450 structures of Ln(III) complexes with these families of ligands. The structures reported contain a wide variety of donor groups appended to the cyclic structure of cyclen or the linear diethylenetriamine unit of H_5_DTPA. This rich source of structural data provides a unique opportunity to rationalize the coordination chemistry of the Ln(III) ions.

The ionic radii of the Ln(III) ions were defined by Shannon in his seminal paper for coordination numbers (CNs) 8 and 9, and in some cases, 6, 7, and 10.^19^ These ionic radii were obtained by analyzing the M–O and M–F distances from oxides and fluorides. Refined values of Shannon’s radii, as well as a new set of ionic radii for uncommon combinations of CNs and oxidation states, were obtained recently using machine learning.^20^ Ionic radii for Ln(III) ions derived from EXAFS data in solution are in good agreement with Shannon’s radii.^21^ In the case of transition metal ions, ionic radii are also affected by the electronic spin as high- and low-spin configurations are characterized by different radii and covalency. As mentioned previously, the Ln(III) ions are hard Lewis acids according to Pearson’s classification, and the Ln(III)-ligand interactions are essentially determined by electrostatic and steric factors. Thus, the CN and coordination polyhedron in a particular Ln(III) complex are mainly dictated by the nature and topology of the ligand and the size of the metal ion. The lanthanide series is characterized by a smooth contraction in ionic radii on increasing atomic number, which amounts to only ∼15%.^22^ As a result, Ln(III) complexes often form an isostructural series of complexes in which the Ln(III)-donor distances progressively decrease across the series. However, abrupt changes in coordination number and coordination polyhedron have also been observed.^23^ Nevertheless, the Ln(III) ions certainly comprise the most coherent family of metal ions across the periodic table in relation to their coordination properties. Recent work suggests that a linear variation of Ln-donor distances across the series is more common than a curved dependence.^24,25^

The calculation of Shannon’s effective ionic radii relies on the assumption that interatomic distances can be approximated as the sum of cation and anion radii, which change with the coordination number. Some authors, who argued that it is doubtful that an unambiguous separation of a bond into a cation and an anion part can be reached, have heavily criticized this assumption.^26^ Indeed, covalence significantly affects interatomic distances, even in Ln(III) compounds.^27^ As a result, ionic radii often do not provide realistic estimates of the size and shapes of ions.^28^ In an attempt to circumvent this problem, some authors derived radii from the distribution of electron density,^29−31^ which is an observable that can be measured experimentally or calculated using quantum mechanical calculations. Nevertheless, ionic radii remain a very useful tool of widespread use by chemists, material scientists, and mineralogists to rationalize structural data. In the case of covalent compounds, bond lengths could be predicted to a surprisingly good accuracy as the sum of two atomic radii, providing the bond is not too ionic, the corresponding coordination numbers are not very different and the concerning bonds do not have multiple bond character (i.e., bonds involving transition metals and halides).^32−34^

**Figure 1:**
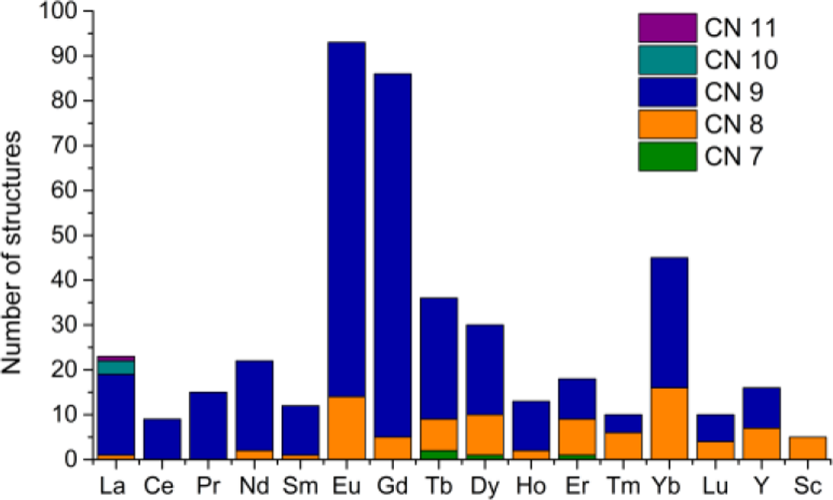
Coordination numbers observed in the X-ray structures of the Ln(III) DOTA^4–^ and DTPA^5–^ derivatives.

From the perspective of coordination chemistry in aqueous media, most Ln(III) complexes display coordination numbers 8 and 9, though some examples of complexes with coordination numbers 7, 10, and even 11 have been described (Figure 1). Two La(III) complexes with DOTA^4–^ derivatives containing amide pendants were found to possess CN 10 in the solid state, where solvent molecules and counterions complete the metal coordination environment.^35,36^ A DTPA-bis-amide La(III) complex with CN 11 was also reported.^37^ All the remaining Ln(III) DOTA^4–^ and DTPA^5–^ derivatives show CNs 8 and 9, with a few exceptions of seven-coordinate complexes. The latter are Tb(III), Dy(III), and Er(III) complexes with cyclen-based ligands that contain bulky phenolate pendant arms.^38,39^ The relative abundance of CN 8 increases across the lanthanide series as the ionic radius of the metal ion decreases, as would be expected. The concept of the coordination number has also been a matter of debate in some cases. For instance, Ln–OH_2_ distances in some Ln(III) DOTA^4–^ derivatives increase along the second half of the lanthanide series as the size of the metal ion contracts,^40^ which led to estimates of noninteger coordination numbers.^41^

A few structures of lanthanide DOTA^4–^ derivatives with oxidation states other than +3 have also been also described. Indeed, four structures of eight-coordinate Eu(II) complexes have been reported. Of these structures, three of them are cyclen derivatives containing phosphonate, phosphinate, or amide pendant arms,^42,43^ while the last structure is a DTPA^5–^ complex.^44^ Recently, the Ce(IV) complexes of DOTA^4–^ and the analogue with methylenephosphonate arms (DOTP^8–^) were also characterized using X-ray diffraction.^45^

In this work, we sought to take advantage of the large body of structural data reported for Ln(III) DOTA^4–^ and DTPA^5–^ derivatives to obtain radii for different donor atom types. These donor radii were obtained from the crystal radii determined by Shannon for CNs 8 and 9, assuming that the Ln(III)-donor distances can be approximated by the sum of Ln(III) and donor radii. We have chosen the set of expanded Shannon’s radii^19,20^ for our study due to its widespread use in coordination chemistry. The donor radii reported here will provide key information to analyze the structures of Ln(III) complexes, particularly to identify particularly strong or weak interactions. This may have very important implications in understanding not only the stereochemical properties of Ln(III) complexes but also their stability and reactivity. For instance, particularly weak Ln(III)–O_water_ interactions, associated with the coordination of a water ligand at a sterically hindered capping position, were correlated to fast water exchange rates,^46,47^ an important parameter to be controlled to optimize the efficiency of MRI contrast agents. Long Ln(III)–O distances involving carboxylate groups were also correlated to fast dissociation rates following the acid-catalyzed mechanism.^48^ We have also extended our analysis to Y(III) and Sc(III), which together with the lanthanides form the rare earths. The coordination chemistry of Y(III) is very similar to that of the lanthanides^49^ and is relevant for the development of radiopharmaceuticals for both imaging and therapy.^50^ The coordination chemistry of Sc(III) with polyaminocarboxylate ligands is currently in its infancy, but interest is growing due to the potential of the ^44^Sc-radionuclide in PET imaging,^51^ and its potential as a diagnostic match of β^–^ emitters ^177^Lu- and ^90^Y-radionuclides.^12^

## Results and Discussion

### Description of the Data Set

The CSD contains crystal data for a large number of lanthanide DOTA^4–^ and DTPA^5–^ derivatives, although the number of hits varies significantly across the lanthanide series. A total of 443 structures of DOTA^4–^ and DTPA^5–^ derivatives containing Ln(III), Y(III), or Sc(III) ions have been analyzed, among which 83% correspond to DOTA^4–^ derivatives. The number of DTPA^5–^ derivatives reported for Gd(III) (31%) is, however, significantly higher than for other lanthanides, a situation that is reflected in the application of these complexes as MRI contrast agents in clinical practice. A few additional structures containing Ln–H and Ln–C bonds have been excluded from this study. The lanthanides with the highest number of hits are Eu (21%), Gd (19%), Yb(10%), and Tb (8%), which reflects the numerous investigations related to the photophysical properties of Eu(III) and Tb(III) complexes and the development of Gd(III)-based contrast agents. These three lanthanides sit at the center of the lanthanide series, with Eu and Tb flanking Gd. Thus, the structural data available are far from being uniformly spread along the series. A significant number of hits were also obtained for Dy(7%). However, very few structures were reported for lanthanides such as Tm (2.3%, 10 hits) and Sc (1.2%, 5 hits, Figure 1).

### Bond Distances

**Figure 2:**
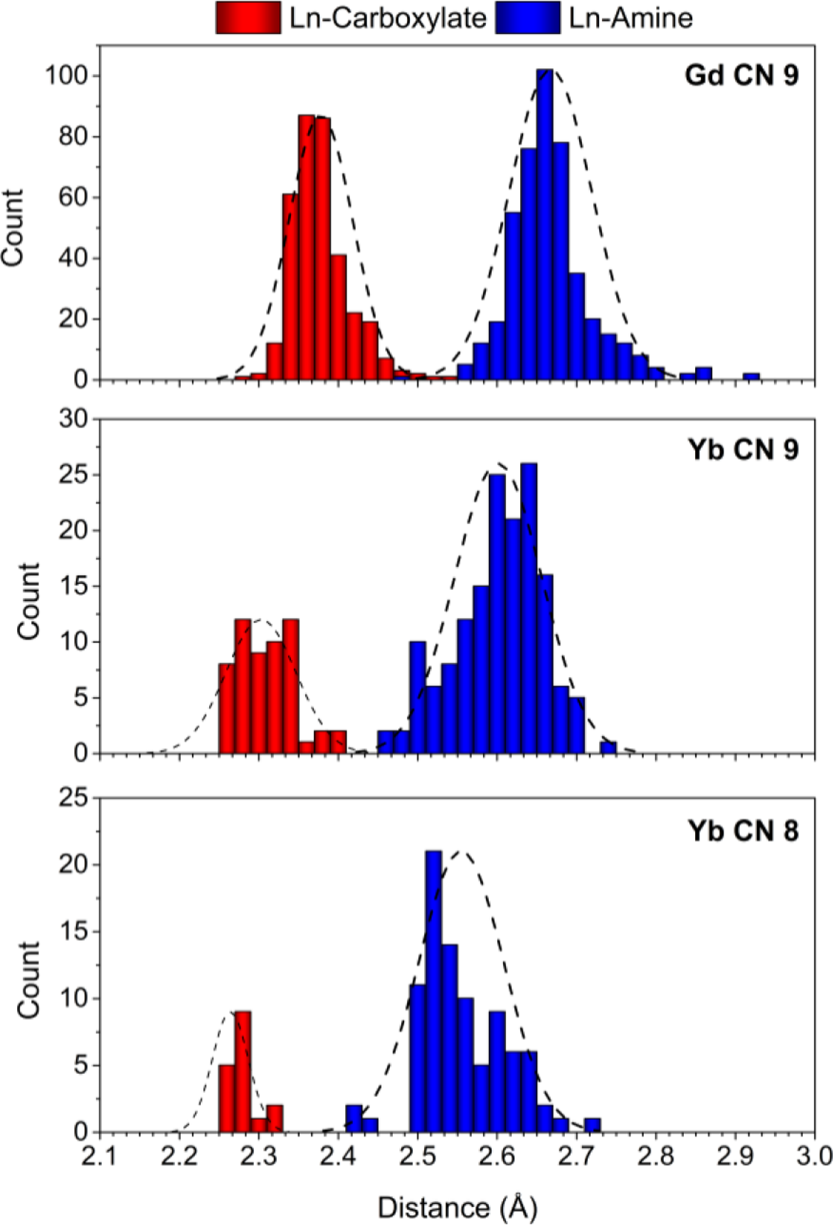
Histograms showing the bond distances involving the Ln(III) ion, carboxylate-O donor atoms, and amine N atoms. Top panel: Gd(III) complexes with CN 9 (347 Gd–O_C_ and 450 Gd–N_A_ distances); central panel: Yb(III) complexes with CN 9 (63 Yb–O_C_ and 155 Yb–N_A_ distances). Low panel: Yb(III) complexes with CN 8 (30 Yb–O_C_ and 89 Yb–N_A_ distances).

O atoms of carboxylate groups (O_C_) and amine N atoms (N_A_) are the most common donor atoms in the family of complexes analyzed here. The Gd–N_A_ and Gd–O_C_ distances for complexes with CN 9 display fairly good Gaussian distributions centered at 2.667 and 2.378 Å, respectively (Figure 2). Thus, Gd–N_A_ distances are consistently longer than Gd–O_C_ ones, as noticed previously by analyzing a small set of complexes.^52^ This can be attributed, at least in part, to the larger covalent radii of N compared to O.^32^ These maxima shift to 2.601 and 2.303 Å for Yb(III) nine-coordinate complexes, which reflects the contraction in bond distances as a result of lanthanide contraction. These shifts of the maxima by ∼0.07 Å are close to that predicted by the corresponding difference in the (revised) Shannon’s radii (0.065 Å). Decreasing the CN from 9 to 8 results in a further shift of the maxima of the Gaussian distributions to 2.555 and 2.264 Å, which represents a contraction of ∼0.05 Å, again close to the variation expected according to the ionic radius (0.057 Å).

The half-width at half height of the Gaussian distributions of Gd–N_A_ and Gd–O_C_ distances amounts to 0.123 and 0.094 Å, respectively. The rather broad distribution of Gd–N_A_ distances is likely related to relatively weak bonds compared with those that involve carboxylate O atoms. This is in line with the lower contribution of amine N atoms compared with carboxylate O atoms to Gd(III) complex stability estimated recently.^10^ The analysis of the electron density at the critical points of the concerned bonds also suggests that Gd–N_A_ bonds are weaker than Gd–O_C_ bonds.^53^ It has been also noted that Ln–N bonds are characterized by rather shallow potential energy surfaces, while Ln-O_C_ bonds display potential energy surfaces with deeper energy minima.^54^ Indeed, some of the Gd–N_A_ distances shown in Figure 2 are particularly long (>2.9 Å),^55^ and one can reasonably associate these distances to weak interactions.

### Calculated Electron Densities

**Figure 3:**
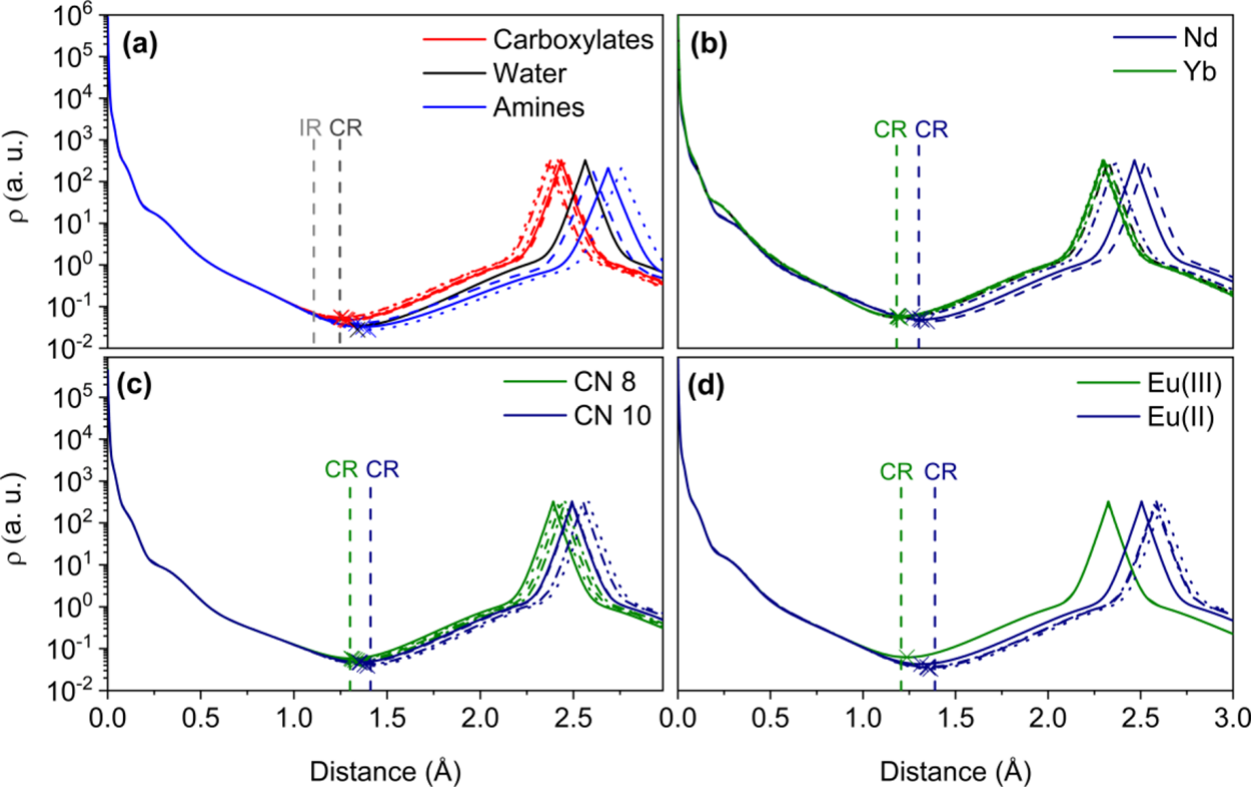
Electron densities (ρ) calculated along the Ln-donor paths for different lanthanide complexes with the metal ion placed at the origin. The position of the donor atom corresponds to the maximum of ρ around 2.5 Å; CPs indicated with crosses: (a) [Gd(DTPA)(H_2_O)]^2–^, CSD code FEPREY; (b) Ln–O_C_ bonds in [Ln(DTPA)(H_2_O)]^2–^, Ln = Nd, Yb, CSD codes CUVZOI and KOLGIB); (c) La–O_AM_ bonds in [La(DOTAM)]^3+^ (CN 8) and [La(DOTAM)(CF_3_SO_3_)(EtOH)]^3+^ (CN 10), CSD codes PIBGOW and PIRSEO; (d) Eu–O_Phos_ bonds in [Eu^III^(DOTP)]^5–^ and [Eu^II^(DOTP)]^6–^, CSD codes AXAMET and ONETEJ. Electron densities were calculated with Multiwfn.^68^

The separation of the Ln-donor distances into contributions of the metal ion and the donor atom should ideally reflect the shape of the electron density (ρ) along the bond axis.^30^ Chemical bonds are characterized by the presence of (3,–1) critical points (CPs) at coordinate *r*_c_, where the first derivative of the electron density vanishes (∇ρ(*r*_c_) = 0). At this point, the electron density is a minimum along the internuclear axis and a maximum in the plane perpendicular to that axis. A (3,–1) CP is situated on the interatomic surface separating the basins of two neighboring atoms.^56^ Thus, prior to the estimation of donor radii, we analyzed the electron density along the Ln-donor axes of the selected systems (Figure 3). For this purpose, we used relativistic all-electron DFT calculations^57,58^ using the DKH2^59^ method and the wB97X-D3BJ functional,^60,61^ which incorporates empirical dispersion.^62^ Basis sets developed specifically for relativistic calculations were used throughout^63,64^ (see computational details in the Supporting Information).

The highest values of ρ are centered on the nuclear positions of the metal ion and, to a lesser extent, on the donor atom. This reflects that most of the electron density sits around the nuclear positions, which act as attractors in the gradient vector field ∇ρ.^65^ The value of ρ then decreases along the internuclear axis and reaches a minimum, which corresponds to the position of the (3,–1) CPs. Figure 3a compares the values of ρ along the different Gd-donor bonds in [Gd(DTPA)(H_2_O)]^2–^. One can notice that the five Gd–O_C_ distances are quite similar, while more significant differences are observed for the three Gd–N_A_ distances. All Gd–O_C_ bonds display very similar positions of the CPs at distances of ∼1.275 Å from the metal ion. The minimum of the electron density deviates significantly from the ionic radius (IR) of Gd(III) (1.107 Å for CN 9).^20^ However, the position of the CPs agrees rather well with the crystal radii (CR) of Gd(III) (1.247 Å for CN 9). CR were introduced by Fumi^66^ and are considered to correspond more closely to the size of an atom (or ion) in a solid, as pointed out by Shannon in his seminal paper.^19^ CR differ from IR by a constant factor, with the former being 0.14 Å longer than the latter. IR were introduced to comply with the 1.40 Å radius for O^2–^ (CN = 6) proposed by Pauling,^67^ while CR use a radius of 1.26 Å for the anion. The positions of the critical points for Gd–N_A_ and Gd–O_W_ bonds (O_w_, oxygen atom of a water molecule) are further apart from the Gd(III) ion compared with Gd–O_C_ bonds, deviating from the CR by ca. 0.14 Å. The values of ρ at the CPs are lower for Gd–N_A_ than Gd–O_C_ bonds, as observed previously.^53^ It is worth noting that calculations performed at the NEVPT2 level provide nearly identical ρ values along the Gd-donor axes (Figure S1).

A comparison of the electron densities along the Ln–O_C_ bonds for [Ln(DTPA)(H_2_O)]^2–^ complexes (Figure 3b) shows that the position of the CPs shifts toward the Ln(III) ion on proceeding across the series from Nd to Yb, as expected due to the lanthanide contraction. This displacement of the CPs averages 0.129 Å for the five Ln–O_C_ bonds, a value that is very similar to the shortening observed for the bond distances, at an average of 0.156 Å, and the difference in CR (0.120 Å). This suggests that the Ln–O_C_ bonds can be broken down into contributions of the metal ion radius and the radius of the donor atom. The positions of the CPs are in very good agreement with those marked by the CR. Similar conclusions can be drawn by analyzing the Ln–N_A_ bonds (Figure S2).

IR and thus CR are defined as specific coordination numbers. To test whether the shape of ρ across the bond path supports this assumption, we carried out calculations on the [La(DOTAM)]^3+^ and [La(DOTAM)(CF_3_SO_3_)(EtOH)]^3+^ complexes, in which the La(III) ions display CNs 8 and 10, respectively (Figure 3c). Calculations evidence that the CPs characterizing the La–O_AM_ bonds (O_AM_ = amide oxygen atom) are indeed affected by the CN, with the minima obtained for CN 10 (∼1.371 Å) and 8 (∼1.320 Å), correlating well with the corresponding CR (∼1.411 and 1.301 Å, respectively).

The size of a metal ion is obviously affected by its charge. The coordination chemistry of the rare earths is largely dominated by the trivalent oxidation state. However, complexes of Ce(IV) and Eu(II) with DOTA^4^^–^ and DTPA^5^^–^ derivatives were characterized by using X-ray diffraction. The electron densities calculated for the Eu–O_PHOS_ bonds in [Eu^III^(DOTP)]^5–^ and [Eu^II^(DOTP)]^6–^ show that the position of the CPs shifts according to what would be expected considering the CR of Eu(II) (1.389 Å) and Eu(III) (1.206 Å) and the average Eu–O_PHOS_ distances (2.573 and 2.325 Å, respectively). A similar conclusion can be reached by analyzing the [Ce^III^(DOTA)(H_2_O)]^−^ and [Ce^IV^(DOTA)(H_2_O)] systems (Figure S3).

The results of our calculations support that the Ln-donor bonds can be indeed divided into a contribution from the metal ion, which can be approximated by CR, and a contribution from the donor atom. This approach has of course some limitations, as the size of the metal ion determined by the electron density changes depending on the nature of the donor atom, as illustrated in Figure 3a. However, this effect is still relatively small (∼0.12 Å) compared with the overall bond distance. We also note that the weak Ln-N_A_ bonds are characterized by rather shallow minima, so that the position of the CP and that marked by the CR are characterized by similar values of ρ. We also note that the coordination geometry is likely to have a minor effect on the position of the CPs, as evidenced by the results obtained for the capped square antiprismatic (SAP) and capped twisted square antiprismatic (TSAP) isomers of [Ce^III^(DOTA)(H_2_O)]^−^ (Figure S4).

### Donor Radii

**Figure 4:**
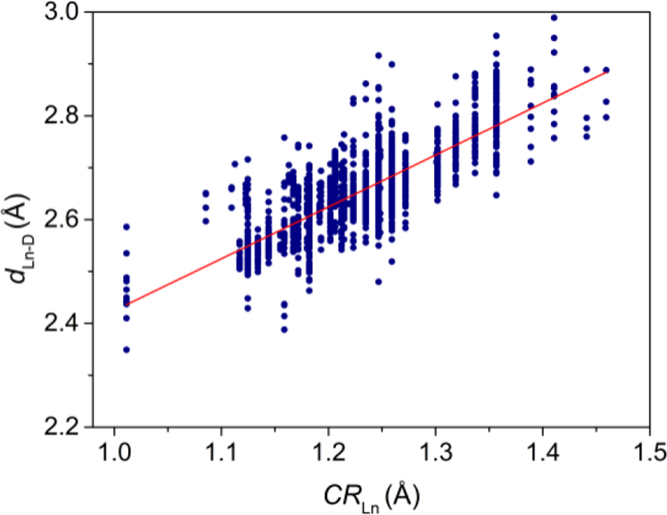
Plot according to eq 1 of bond distances (*d*_Ln–D_) involving amine N atoms versus crystal radii.

Having shown that the Ln-donor distances can be reasonably separated into metal ion and donor contributions, we sought to estimate radii for different donor groups commonly present in DOTA^4–^ and DTPA^5–^ derivatives using the following expression:
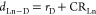
1where *d*_Ln–D_ represents the Ln^3+^-donor distance, *r*_D_ is the radius of a given donor atom, and CR_Ln_ is the crystal radius of the Ln^3+^ ion. In our analysis, we used CR obtained after adding a value of 0.14 Å to the refined Shannon’s radii reported recently. Thus, a plot of *d*_Ln–D_ versus CR_Ln_ should provide a linear trend whose intercept corresponds to *r*_D_ when the slope is fixed to 1. Figure 4 provides examples of these plots as well as the linear fits of the data. Since CR take different values depending on the coordination number, one needs to establish the coordination number a priori in each case. For this work, we assumed that the coordination number is defined by the number of donor atoms within 3.0 Å distance from the metal center. This rather long threshold was chosen to incorporate all donor atoms involved in metal ion coordination into the analysis, including those providing weak interactions, to obtain a truly representative value of the donor radius. All donor atoms within this threshold form a bond with the lanthanide according to Bader’s postulate that a bond between a pair of atoms exists if a (3,–1) CP is present along the internuclear axis. Thus, the CN of the Ln(III) ion equals the number of (3,–1) CPs in its surroundings. Calculations performed on the [Er(DOTMA)(H_2_O)]^−^ complex, which contains a long Er–OH_2_ distance of 2.732 Å, confirm the presence of a (3,–1) CP along the Er–O internuclear axis (Figure S5).

The advantage of this analysis is that the *r*_D_ values are obtained from the simultaneous fit of bond distances observed for all rare-earth complexes, excluding radioactive *Pm*. Thus, this approach provides a clear advantage over the simple analysis of bond distances for a particular Ln^3+^ ion, Sc^3+^ or Y^3+^. Indeed, this approach allows for accumulating enough bond distance data for a specific donor atom, even if none or a few values are available for specific Ln^3+^ ions. Furthermore, the fitted *r*_D_ values are universally valid for all earth rare cations in any oxidation state, with the only limitation being having reliable CR_Ln_ data. This provides a very useful tool to analyze complexes with metal ions (i.e., Tm^3+^ and Sc^3+^) or oxidation states (Eu^2+^ and Ce^4+^), for which limited structural data are currently available.

Figure 4 presents an example of a fit according to eq 1 for amine N atoms. The linear trend evidences that on average, Ln-donor distances decrease with the decreasing CR of the rare earth, as expected due to the lanthanide contraction. The points in this plot are aligned in 38 vertical lines, each representing a value of CR_Ln_, which varies depending on the rare-earth element, its oxidation state, and coordination number. The lowest value of CR_Ln_ corresponds to Sc^3+^ with CN = 8 (1.011 Å), while the upper limit is given by La^3+^ with CN = 11 (1.459 Å). Linear plots for other donor atoms are shown in Figures S6–S13). The linear fits of the data afforded the *r*_D_ values listed in Table 1.

**Table 1:** Donor Radii (*r*_D_) and Line Widths (Δ*d*_1/2_) and Centers (*x*_0_) of the Gaussian Distributions for Rare-Earth Complexes^a^

	*r*_D_ (Å)^c^	Δ*d*_1/2_ (Å)	*x*_0_ (Å × 10^3^)
N_AM_	1.425 ± 0.001	0.097	–7.58
O_C_	1.132 ± 0.001	0.071	–3.62
O_A_	1.130 ± 0.002	0.062	–10.2
Cl	1.451 ± 0.014	b	b
F	0.915 ± 0.008	b	b
O_PO3_	1.114 ± 0.004	0.067	–1.82
O_PRO2_	1.106 ± 0.002	0.077	–0.44
N_PY_	1.329 ± 0.003	0.053	–4.71
O_Tf_	1.183 ± 0.007	b	b
O_W_	1.231 ± 0.004	0.123	–26.3
O_OH_	1.157 ± 0.006	0.058	–2.51

a N_AM_, amine nitrogen; O_C_, carboxylate oxygen; O_A_, amide oxygen; Cl, chloride anion; F, fluoride anion; O_PO3_, phosphonate oxygen; O_PRO2_, phosphinate oxygen; N_PY_, pyridine nitrogen; O_Tf_, Triflate oxygen; O_W_, water oxygen; O_OH_, alcohol oxygen.

b The limited number of data available prevented accurate Gaussian fitting.

c These values can be also used in combination with IR by adding 0.14 Å to the corresponding donor radius. The errors correspond to the standard deviations of the linear fits (see text).

**Figure 5:**
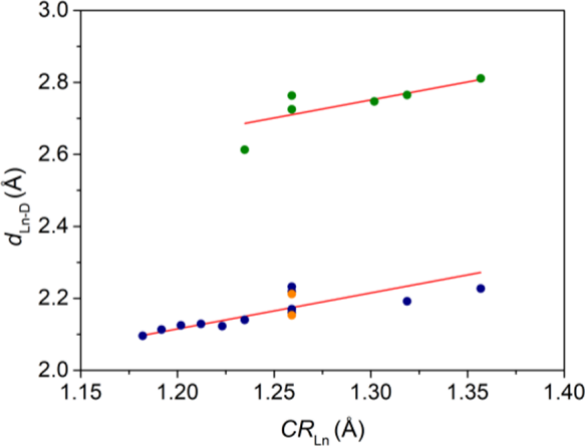
Plots according to eq 1 of bond distances (*d*_Ln–D_) involving chloride (green circles) and fluoride (blue circles). The orange circles correspond to distances erroneously assigned to Ln–Cl bonds instead of Ln–F bonds (see test).

The linear plot obtained for chloride deserves further comment (Figure 5). In spite of the rather limited number of data available for chloride, the observed distances fall within the approximate range of 2.6–2.8 Å, with the exception of two [Eu(L^py^)Cl]^3+^ complexes reported by Wada,^69^ in which Eu–Cl distances of ∼2.2 Å were reported (orange circles in Figure 5, L^py^ = 1,4,7,10-tetrakis(pyridin-2-ylmethyl)-1,4,7,10-tetraazacyclododecane, Chart 1). These Eu–Cl distances are, however, within the range expected for Eu–F terminal bonds. The same work reports complexes with Eu–Cl distances of 2.73–2.75 Å. The two complexes with extremely short Eu–Cl bonds were isolated and crystallized as salts of PF_6_^–^ and BF_4_^–^, which made us suspect that a F atom was assigned incorrectly to a chloride atom. It is worth noting that terminal Ln–F bonds are significantly shorter than bridging F–Ln–F bonds.^64^ Thus, the value of *r*_D_ reported in Table 1 (and Figure 5) for fluoride was obtained from data for Ln–F bonds involving terminal fluoride coordination.

**Figure 6:**
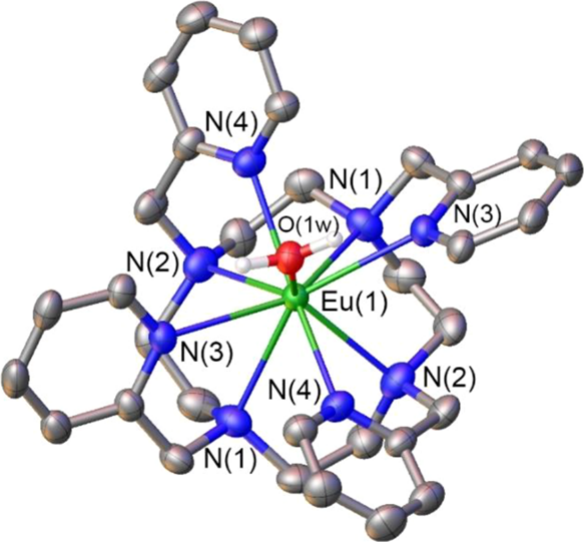
Structure of the [Eu(L^py^)(H_2_O)]^3+^ cation present in the crystals of [Eu(L^py^)(H_2_O)](PF_6_)_3_·2H_2_O.

The extremely short Ln–Cl distances reported by Wada prompted us to prepare and crystallize the complex. Both the ligand and the Eu(III) complex were prepared following reported procedures,^69,70^ using the chloride salt. Addition of KPF_6_ to an aqueous solution of the complex afforded single crystals with the formula [Eu(L^py^)(H_2_O)](PF_6_)_3_·2H_2_O. The [Eu(L^py^)(H_2_O)]^3+^ cation contains a coordinated water molecule with a Eu–O_W_ distance of 2.378(7) Å, rather than a chloride or fluoride anion (Figure 6). This distance is somewhat shorter than that estimated from the values of *r*_D_ and crystal radius (2.49 Å), which is expected considering the positive charge of the complex.^71^ Interestingly, the crystal data can also be refined satisfactorily assuming that the ligand present in the apical position is a chloride or a fluoride anion. The refinement of the structure with these incorrect atom assignments yields very similar *R*1 and w*R*2 factors (Table S2), and no major (A type) alerts in the checkcif reports. Thus, most likely, the structures reported by Wada contain a Eu–F rather than a Eu–Cl bond.

**Figure 7:**
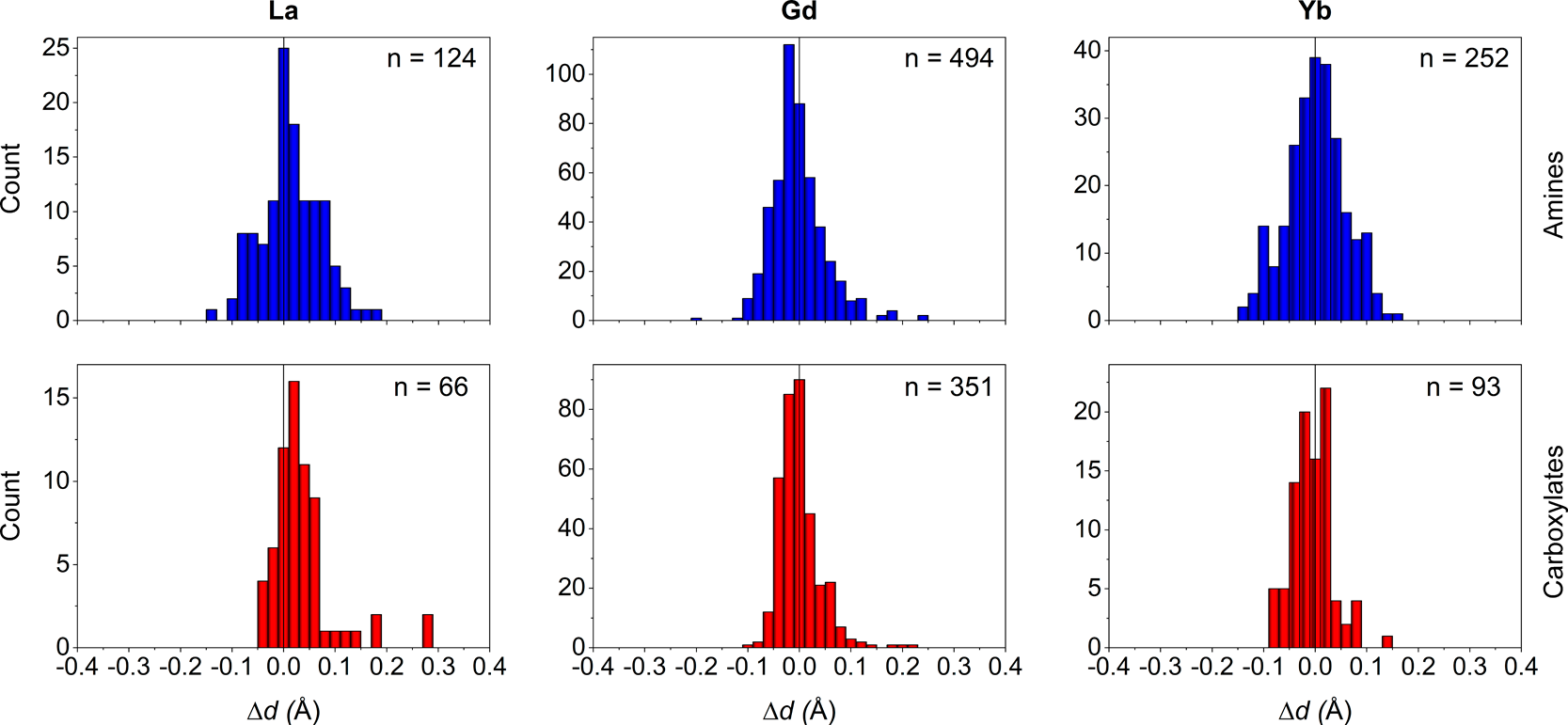
Histograms showing the differences between experimental and calculated bond distances involving the Ln(III) ion and carboxylate O donor atoms and amine N atoms (Δ*d* = *d*^exp^ – *d*^cal^, with *d*^cal^ = *r*_D_ + CR_Ln_).

Figure 7 shows the difference between experimental and calculated bond distances (Δ*d*) of amine and carboxylate donor atoms obtained for three rare-earth cations representative of large (La^3+^), medium (Gd^3+^), and small (Yb^3+^) cations. The values of Δ*d* display rather good Gaussian distributions with maxima close to Δ*d* = 0. The fact that the maximum of the Gaussian distribution does not drift across the lanthanide series provides confidence in the reliability of our approach. One can notice that Ln–O_C_ bonds are characterized by narrower distributions than Ln–N_A_ bonds, as mentioned above. Most of the calculated bond distances fall within the −0.1 Å < Δ*d* < 0.1 Å window (<90%). Furthermore, the bonds with the largest deviations are associated with unusual coordination features. For instance, the La–O_C_ bonds characterized by Δ*d* = 0.271 Å correspond to a dinuclear La^3+^ complex, with CN 11 and carboxylate bridges coordinating through a μ^2^-η^2^:η^1^ mode.^37,72^ The La–O_C_ distances with Δ*d* = ∼ 0.17 Å also correspond to bridging carboxylate groups coordinating in a μ^2^-η^2^ mode.^73^

**Figure 8:**
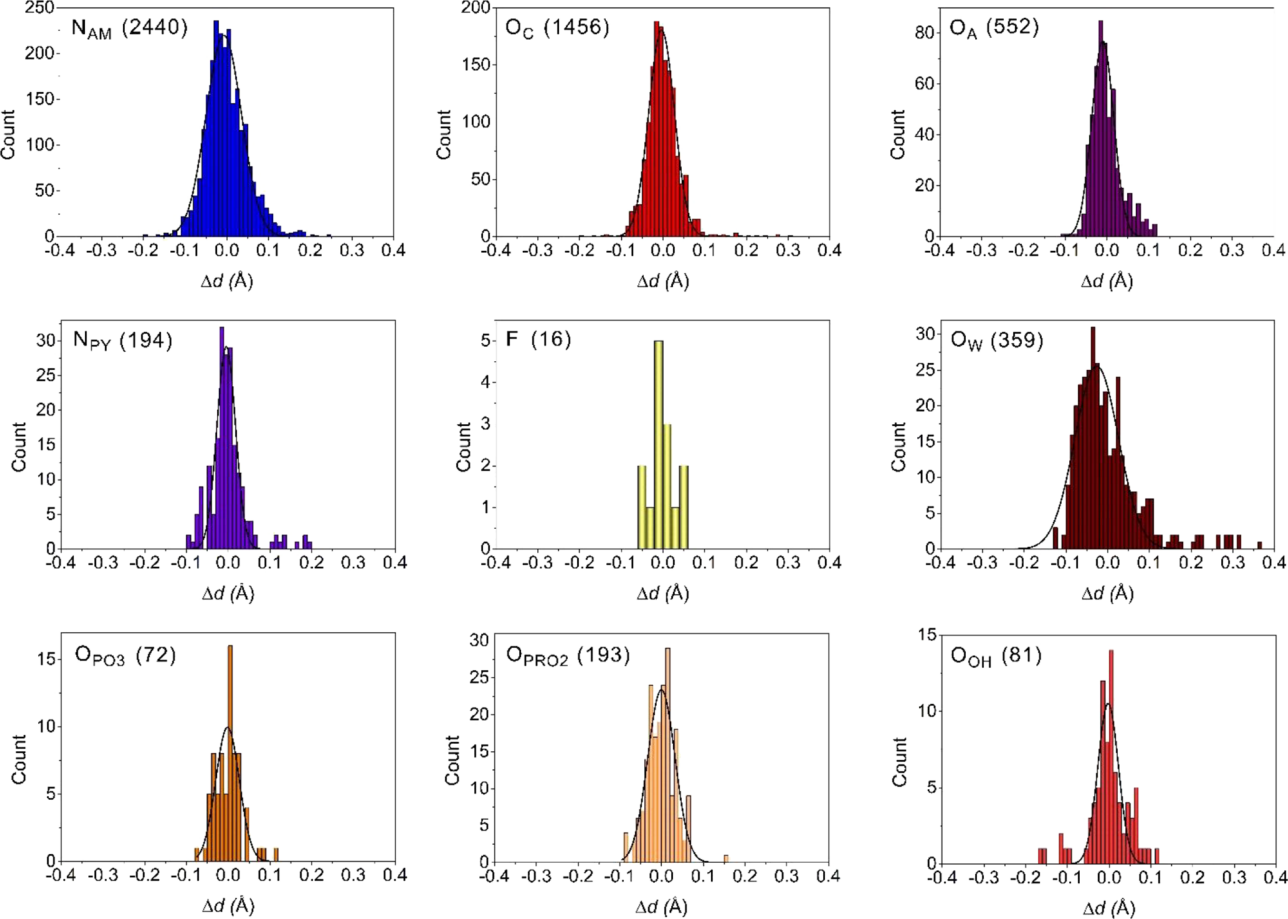
Histograms showing the differences between experimental and calculated bond distances involving the rare-earth ion and different donor atoms (Δ*d* = *d*^cal^ – *d*^exp^, with *d*^cal^ = *r*_D_ + CR_Ln_). Histograms were constructed from data obtained for all rare-earth cations. The numbers within parentheses indicate the number of data points.

The values of Δ*d* obtained for all rare-earth cations and nine different donor atoms are shown in Figure 8. The histograms were subsequently fitted to Gaussian functions, which provided the fitted values of the center (*x*_0_) and the width at half height (Δ*d*_1/2_). For some donor atoms (F, Cl, and O_Tf_), the limited number of data prevented accurate Gaussian fitting. All fitted Gaussian functions show very small *x*_0_ values, indicating that the Gaussian functions are centered at *d*_Ln–D_ ∼ *r*_D_ + CR_Ln_ (Δ*d* ∼ 0). The absolute values of *x*_0_ are generally equal to or lower than 0.01 Å, with the exception of the Ln–O_w_ bonds, for which *x*_0_ = −0.026 Å. The Ln–O_w_ bonds are also characterized by a rather broad distribution, with Δ*d*_1/2_ = 0.123 Å (Table 1).

We note that the histograms are not perfectly symmetrical, showing a hump on the positive side. This implies that the number of distances with positive deviations (*d*_Ln–D_ > *r*_D_ + CR_Ln_) exceeds those with negative Δ*d* values. The shape of the histograms resembles, to a certain extent, that of a potential energy curve associated with a bond distance, which is often described by a Morse function.^74^ This suggests that shortening the Ln-donor distance beyond the value of *x*_0_ provokes a steeper increase in energy than weakening the Ln-donor interaction. The asymmetry of the histogram is particularly evident in the Ln–O_W_ bonds, for which positive deviations of Δ*d* of up to 0.35 Å are observed (Figure 8). This is expected, as most donor atoms are incorporated into the macrocyclic or linear scaffold of the ligand. In this situation, the spread of Δ*d* values is reduced, as increasing a *d*_Ln–D_ distance above a certain value will weaken the interaction with the neighboring donor atoms. Water generally acts as a monodentate ligand, and thus, the Ln–O_W_ distance can vary significantly without a significant energy cost. In fact, rare-earth complexes present in solution with different hydration numbers are rather common.^75−78^ Extremely long Ln–O_W_ distances in DOTA derivatives have been also correlated with fast exchange rates of the coordinated water molecule.^40,41^ This has been explained by the fact that a weak Ln–O_W_ interaction reduces the energy cost to reach the transition state responsible for the dissociative water exchange mechanism.^47^ Water molecules occupying sterically demanding capping positions in the coordination polyhedron display particularly fast water exchange rates, a phenomenon that has been defined as the labile capping bond effect.^46,79^ The large steric demand of capping positions is also evidenced in the structures of nine-coordinate Eu(III) and Er(III) DO3A derivative complexes containing a phenanthroline pendant.^80^ The N atom of the phenanthroline unit at the capping position is characterized by long Ln–N distances with Δ*d* = +0.185 and 0.197 Å for Eu(III) and Er(III), respectively. Conversely, the pyridyl donor atom at the upper plane of the SAP coordination polyhedron is characterized by a short distance (Δ*d* = −0.066 and −0.092 Å for Eu(III) and Er(III), respectively).

The values of *r*_D_ obtained from the linear fits of the data according to eq 1 show that donor radii vary significantly for the different donor atom types. Among the groups containing O donor atoms, phosphinate and phosphonate atoms present the smallest *r*_D_ values, followed closely by carboxylate and amide atoms, which present identical *r*_D_ values within statistical error. Alcohol donor groups present a slightly larger *r*_D_ value than carboxylates and amides, while water is characterized by the largest *r*_D_ value among all the donor groups considered here. An oxygen atom of a triflate anion presents an *r*_D_ value intermediate between those of water and alcohol O atoms. The *r*_D_ values involving N donor atoms are higher than those of Ln-O bonds, as would be expected according with the covalent radii of O and N.^32^ The same reason explains the very short *r*_D_ value obtained for fluoride and the long *r*_D_ value obtained for chloride. Among the N donor atoms considered here, amine N atoms are characterized by a significantly longer *r*_D_ value than that of pyridine, with the latter also showing a narrower distribution.

A relatively small number of X-ray structures analyzed here contained protonated carboxylate groups (13 data points), which display Δ*d* values in the range of 0.010–0.115 Å.^81−85^ Thus, protonation of a carboxylate group provokes only a slight increase of the Ln–O_c_ bond distances. Protonation of coordinated phosphonate groups does not have any significant effect (−0.072 < Δ*d* < 0.044 Å).^45,86,87^

It is worth noting that the *r*_D_ values do not correlate with the contributions of these donor groups to complex stability. For instance, we have estimated recently that phosphonate groups contribute with Δlog *K* = 5.0 units to the stability of a Gd^3+^ complex, while the contribution of a phosphinate group is only 1.9 log *K* units.^10^ However, both donor groups display virtually identical *r*_D_ values.

The *r*_D_ values reported here allow for the estimation of a typical Ln-donor distance for any rare-earth complex. This is very useful, as there is no set of radii available for donor atoms that can provide good estimates of lanthanide-donor bond distances. The crystal CR reported by Shannon do not provide good estimates of Ln(III)-donor distances in polyaminopolycarboxylate complexes. Furthermore, Shannon defined different CR for the oxide anion depending on the coordination number (1.21–1.28 Å).^19^ The value of 1.21 Å (for CN 2) is 0.08 Å longer than the donor radius of a carboxylate oxygen atom (1.132 Å). Differences even increase when taking CR for higher coordination numbers. The deviation is significantly larger for a phosphonate or phosphinate (up to 0.1 Å). This is a huge difference, as it roughly corresponds to the distance between the center and the tail of the Gaussian distribution (Figure 8). The situation is not better when van der Waals radii are used for neutral donors. Bondi’s radius^88^ for a N atom of 1.55 Å is significantly larger than the donor radius estimated in our work for an amine donor (1.425 Å) and deviates even more from that of a pyridyl donor (1.329 Å). The situation worsens for neutral O atoms, as the donor radii of amide (1.130 Å) and water (1.231 Å) differ by 0.1 Å, and the van der Waals radius of O is much larger (1.52 Å).^88,89^ Thus, it is clear that there is currently no reliable set of radii that allows for the calculation of Ln-donor distances with reasonable accuracy.

In order to test the usefulness of donor radii to predict bond lengths in lanthanide complexes based on platforms other than H_4_DOTA and H_5_DTPA, the bond distances of complexes based on the 18-membered macrocycle PYAN (i.e., H_4_PYTA, Chart 1) were analyzed.^90−94^ These systems were chosen because of the presence of pyridine groups in the macrocyclic backbone, the high coordination numbers observed for most rare-earth complexes (typically CN 10), and the availability of structures for many of the rare-earths. A plot of the measured bond distances against the distances calculated using the donor radii (Figure S14) shows a reasonably good distribution around the identity function.

## Conclusions

This work has established a straightforward approach to derive donor radii for complexes of rare-earth cations, providing a tool to estimate Ln-donor distances (including Y and Sc) regardless of the nature of the rare-earth cation and its oxidation state. Our approach relies on separating the Ln-donor distances into contributions from the cation, approximated by the CR, and the donor atom. Theoretical calculations provide support for this approach, at least for complexes with polyaminopoly(carboxylate) ligands used for stable complexation in aqueous media. The rare-earth cations are particularly well-suited for the analysis presented here, as they represent the most coherent series of elements within the periodic table in terms of their chemical properties.

The work presented in this contribution provides a rich source of data that will be very helpful in analyzing the structural data of rare-earth complexes. For instance, particularly strong or weak interactions can be easily identified, providing foundations for the interpretation of thermodynamic and kinetic data. Additionally, these radii can be very helpful in crystallographic analysis to prevent incorrect atom assignments. The approach presented here can be easily applied to other donor groups, which were excluded here due to the scarcity of data for these donors in our data set (i.e., phenol, ether oxygen atoms, etc.). Historically, IR have served as a very useful tool for geochemists, mineralogists, chemists, and material scientists to rationalize structural data. However, our results indicate that CR provide a better estimate of lanthanide ion size than IR. Therefore, we recommend the use of CR for the rationalization of structural data of Ln(III) compounds and possibly of other metal ions.

## Supporting Information Available

Computational details, crystallographic data (CCDC 2266985), additional plots showing calculated electron densities, linear fits according to eq 1, and bond distances used in this work with CSD codes (PDF)
