# Supplementary material for: Donor Radii in Rare-Earth Complexes
Source: Inorg Chem. 2023 Oct 2;62(41):17030–40. doi: 10.1021/acs.inorgchem.3c03126 (PMC10583196; doi:10.1021/acs.inorgchem.3c03126)
Supplement: Supplementary file 1 — ic3c03126_si_001.pdf [file ic3c03126_si_001.pdf]

Supporting Information for:

Donor Radii in Rare-Earth Complexes

Charlene Harriswangler[a], Juan C. Frías[b]*, M. Teresa Albelda[c], Laura Valencia[d], Enrique García-España[e], David Esteban-Gómez[a], and Carlos Platas-Iglesias[a,]*

[a] Universidade da Coruña, Centro Interdisciplinar de Química e Bioloxía (CICA) and Departamento de Química, Facultade de Ciencias, 15071, A Coruña, Galicia, Spain

[b] Departamento de Ciencias Biomédicas, Universidad Cardenal Herrera-CEU, CEU Universities, 46115 Valencia, Spain

[c] Departamento de Química Inorgánica, Universidad de Valencia, C/Dr. Moliner 50, 46100 Burjasot, Valencia, Spain.

[d] Departamento de Química Inorgánica, Facultad de Ciencias, Universidade de Vigo, As Lagoas, Marcosende, 36310 Pontevedra, Spain

[e] Instituto de Ciencia Molecular (ICMol), Departamento de Química Inorgánica, Universidad de Valencia, 46980 Paterna, Spain

Email: carlos.platas.iglesias@udc.es (C. P.-I.)
Email: juan.frias@uchceu.es (J. C. F.)

Computational details 3

Crystal structure determination 3

References 3

Table S1: Crystal data and structure refinement for **[Eu(L^{py})(H$_2$O)](PF$_6$)$_3$·2H$_2$O** 5

Table S2: Crystal structure refinement agreement factors obtained for correct ([Eu(L^{py})(H$_2$O)](PF$_6$)$_3$·2H$_2$O) and incorrect atom assignments. 5

Figure S1: Electron densities (ρ) along the Gd-donor paths for [Gd(DTPA)(H$_2$O)]$^{2-}$ obtained using relativistic DFT (wB97X-D3BJ) and NEVPT2 calculations. The two methods provide nearly identical ρ values. CSD code FEPREY. 6

Figure S2: Electron densities (ρ) along the Ln-N$_A$ paths for [Nd(DTPA)(H$_2$O)]$^{2-}$ and [Yb(DTPA)(H$_2$O)]$^{2-}$ obtained using relativistic DFT (wB97X-D3BJ). CSD codes CUVZOI and KOLGIB, respectively. CPs are indicated with crosses. 6

Figure S3: Electron densities (ρ) calculated along the Ce-O$_C$ paths for [Ce^{IV}(DOTA)(H$_2$O)] and [Ce^{III}(DOTA)(H$_2$O)]$^-$ using relativistic DFT (wB97X-D3BJ). CSD codes JEZREP and LUQBII, respectively. CPs are indicated with crosses. 7

Figure S4: Electron densities (ρ) calculated along the Ce-O$_C$ paths for the SAP and TSAP isomers of [Ce^{III}(DOTA)(H$_2$O)]$^-$ using relativistic DFT (wB97X-D3BJ). CSD codes ETIHIB and LUQBII, respectively. CPs are indicated with crosses. 7

Figure S5: Electron density (ρ) calculated along the Er-O_W path for $[Er(DOTMA)(H_2O)]^-$ using relativistic DFT (wB97X-D3BJ). CSD code LOLQEL. CP indicates the position of the (3,-1) critical point, while CR denotes crystal radius.. 8

Figure S6: Plot of bond distances (d_{Ln-D}) versus *CR* involving carboxylate oxygen atoms (O_C)....................... 8

Figure S7: Plot of bond distances (d_{Ln-D}) versus *CR* involving amide oxygen atoms (O_A). 9

Figure S8: Plot of bond distances (d_{Ln-D}) versus *CR* involving phosphonate oxygen atoms (O_{PO3}). 9

Figure S9: Plot of bond distances (d_{Ln-D}) versus *CR* involving phosphinate oxygen atoms (O_{PRO2}). 10

Figure S10: Plot of bond distances (d_{Ln-D}) versus *CR* involving pyridine nitrogen atoms (N_{PY}). 10

Figure S11: Plot of bond distances (d_{Ln-D}) versus *CR* involving triflate oxygen atoms(O_{Tf}).......................... 11

Figure S12: Plot of bond distances (d_{Ln-D}) versus *CR* involving water oxygen atoms (O_W). 11

Figure S13: Plot of bond distances (d_{Ln-D}) versus *CR* involving alcohol oxygen atoms (O_{OH}). 12

Figure S14: Plot of experimental versus calculated bond distances for H_4PYTA derivatives. The solid line corresponds to the identity function. 12

Table S3: Bond distances, position of the CPs, Electron density at the bond critical points (au), coordination numbers (CN), crystal radii and CSD codes.. 13

Table S4: CSD codes and bond distances (Å) observed in X-ray structures of lanthanum complexes. 14

Table S5: CSD codes and bond distances (Å) observed in X-ray structures of cerium complexes............... 17

Table S6: CSD codes and bond distances (Å) observed in X-ray structures of praseodymium complexes. . 19

Table S7: CSD codes and bond distances (Å) observed in X-ray structures of neodymium complexes. 21

Table S8: CSD codes and bond distances (Å) observed in X-ray structures of samarium complexes.......... 24

Table S9: CSD codes and bond distances (Å) observed in X-ray structures of europium complexes. 26

Table S10: CSD codes and bond distances (Å) observed in X-ray structures of gadolinium complexes....... 40

Table S11: CSD codes and bond distances (Å) observed in X-ray structures of terbium complexes. 53

Table S12: CSD codes and bond distances (Å) observed in X-ray structures of dysprosium complexes. 57

Table S13: CSD codes and bond distances (Å) observed in X-ray structures of holmium complexes. 61

Table S14: CSD codes and bond distances (Å) observed in X-ray structures of erbium complexes. 63

Table S15: CSD codes and bond distances (Å) observed in X-ray structures of thulium complexes. 65

Table S16: CSD codes and bond distances (Å) observed in X-ray structures of ytterbium complexes......... 67

Table S17: CSD codes and bond distances (Å) observed in X-ray structures of lutetium complexes. 73

Table S18: CSD codes and bond distances (Å) observed in X-ray structures of yttrium complexes............ 74

Table S19: CSD codes and bond distances (Å) observed in X-ray structures of scandium complexes. 76

Table S20: CSD codes and bond distances (Å) observed in X-ray structures of H_4PYTA derivatives. 77

Computational details

The geometries used for quantum mechanical calculations were obtained from the CSD. The positions of the H atoms were optimized using density functional theory (DFT) with the Gaussian16 program.[1] We selected the wB97XD functional,[2,3] which incorporates empirical dispersion corrections. Relativistic effects were incorporated using the large-core approximation, which incorporates the 4f electrons of the lanthanide in the core, while describing the outer 5s, 5p, 5d, and 6s electrons with a (7s6p5d)/[5s4p3d]-GTO valence basis set.[4] Ligand atoms were described with the Def2-TZVPP basis set.[5] The size of the integration grid was set with the integral=ultrafine keyword.

Electron densities were calculated with the ORCA program system (Program Version 5.0.3)[6,7] using the geometries with optimized H positions. Relativistic effects were incorporated with the second-order Douglas-Kroll-Hess (DKH2)[8,9] approximation with the DKH-def2-TZVPP basis set for ligand atoms and SARC-DKH-TZVPP[10] for the lanthanide. The resolution of identity and chain of spheres (RIJCOSX) approach was used to accelerated the calculations with auxiliary basis sets generated with the Autoaux[11] feature. ORCA calculations were carried out using DFT with the wB97X-D3BJ[2,12] functional, or at the complete active space self-consisted field (CASSCF) level.[13,14] The latter calculations were performed for Gd compounds. The active space included the 7 Gd electrons distributed in the seven metal-based 4f orbitals [CAS(7,7)]. The state-averaged CASSCF wave function included 1 octet and 49 sextet roots. Dynamic correlation was introduced with N-electron valence perturbation theory to second order (FIC-NEVPT2).[15,16]

Crystal structure determination

[Eu(L^{py})(Cl)](Cl)$_2$ was prepared according to the procedure reported by Wada *et al.*[17] **[Eu(L^{py})(Cl)](Cl)$_2$** (5.8 mg, 7.3 µmol) were dissolved in 150 µl of Milli-Q water and 40 µl of a saturated solution of KPF_6 were added. After 24 hours, colorless prismatic crystals of **[Eu(L^{py})(H$_2$O)](PF$_6$)$_3$·2H$_2$O** were obtained.

Compound **[Eu(L^{py})(H$_2$O)](PF$_6$)$_3$·2H$_2$O** was analysed by X-ray diffraction. Crystallographic data and structure refinement parameters are shown in Table S1. Crystallographic data were collected on a Bruker D8 Venture diffractometer with a Photon 100 CMOS detector at 293 K with Mo-Kα radiation (λ = 0.71073 Å) generated by an Incoatec high brillance microfocus source equipped with Incoatec Helios multilayer optics. The APEX3[18] software was used for collecting frames of data, indexing reflections, and the determination of lattice parameters, while SAINT[19] was used for integration of intensity of reflections, and SADABS[20] for scaling and empirical absorption correction. The structure was solved by dual-space methods using the program SHELXT.[21] All non-hydrogen atoms were refined with anisotropic thermal parameters by full-matrix least-squares calculations on F^2 using the program SHELXL-2014.[21] Hydrogen atoms of the compound were inserted at calculated positions and constrained with isotropic thermal parameters. CCDC 2266985 contains the supplementary crystallographic data, which can be obtained free of charge from the Cambridge Crystallographic Data Centre via www.ccdc.ac.uk/data_request/cif.

References

(1) Frisch, M. J.; Trucks, G. W.; Schlegel, H. B.; Scuseria, G. E.; Robb, M. A.; Cheeseman, J. R.; Scalmani, G.; Barone, V.; Petersson, G. A.; Nakatsuji, H.; Li, X.; Caricato, M.; Marenich, A. V.; Bloino, J.; Janesko, B. G.; Gomperts, R.; Mennucci, B.; Hratchian, H. P.; Ortiz, J. V.; Izmaylov, A. F.; Sonnenberg, J. L.; Williams; Ding, F.; Lipparini, F.; Egidi, F.; Goings, J.; Peng, B.; Petrone, A.; Henderson, T.; Ranasinghe, D.; Zakrzewski, V. G.; Gao, J.; Rega, N.; Zheng, G.; Liang, W.; Hada, M.; Ehara, M.; Toyota, K.; Fukuda, R.; Hasegawa, J.; Ishida, M.; Nakajima, T.; Honda, Y.; Kitao, O.; Nakai, H.; Vreven, T.; Throssell, K.; Montgomery Jr., J. A.; Peralta, J. E.; Ogliaro, F.; Bearpark, M. J.; Heyd, J. J.; Brothers, E. N.; Kudin, K. N.; Staroverov, V. N.; Keith, T. A.; Kobayashi, R.; Normand, J.; Raghavachari, K.; Rendell, A. P.; Burant, J. C.; Iyengar, S. S.; Tomasi, J.; Cossi, M.; Millam, J. M.; Klene, M.; Adamo, C.; Cammi, R.; Ochterski, J. W.; Martin, R. L.; Morokuma, K.; Farkas, O.; Foresman, J. B.; Fox, D. J. Gaussian 16 Rev. C.01, 2016.

(2) Chai, J.-D.; Head-Gordon, M. Long-Range Corrected Hybrid Density Functionals with Damped Atom–Atom Dispersion Corrections. *Phys. Chem. Chem. Phys.* **2008**, *10* (44), 6615–6620. https://doi.org/10.1039/b810189b.

(3) Chai, J.-D.; Head-Gordon, M. Systematic Optimization of Long-Range Corrected Hybrid Density Functionals. *The Journal of Chemical Physics* **2008**, *128* (8), 084106. https://doi.org/10.1063/1.2834918.
(4) Dolg, M.; Stoll, H.; Savin, A.; Preuss, H. Energy-Adjusted Pseudopotentials for the Rare Earth Elements. *Theoret. Chim. Acta* **1989**, *75* (3), 173–194. https://doi.org/10.1007/BF00528565.
(5) Weigend, F.; Ahlrichs, R. Balanced Basis Sets of Split Valence, Triple Zeta Valence and Quadruple Zeta Valence Quality for H to Rn: Design and Assessment of Accuracy. *Phys. Chem. Chem. Phys.* **2005**, *7* (18), 3297–3305. https://doi.org/10.1039/b508541a.
(6) Neese, F. The ORCA Program System. *WIREs Comput Mol Sci* **2012**, *2* (1), 73–78. https://doi.org/10.1002/wcms.81.
(7) Neese, F. Software Update: The ORCA Program System, Version 4.0. *WIREs Comput Mol Sci* **2018**, *8* (1), e1327. https://doi.org/10.1002/wcms.1327.
(8) Barysz, M.; Sadlej, A. J. Two-Component Methods of Relativistic Quantum Chemistry: From the Douglas–Kroll Approximation to the Exact Two-Component Formalism. *Journal of Molecular Structure: THEOCHEM* **2001**, *573* (1–3), 181–200. https://doi.org/10.1016/S0166-1280(01)00542-5.
(9) Reiher, M. Douglas–Kroll–Hess Theory: A Relativistic Electrons-Only Theory for Chemistry. *Theor Chem Acc* **2006**, *116* (1–3), 241–252. https://doi.org/10.1007/s00214-005-0003-2.
(10) Pantazis, D. A.; Neese, F. All-Electron Scalar Relativistic Basis Sets for the Lanthanides. *J. Chem. Theory Comput.* **2009**, *5* (9), 2229–2238. https://doi.org/10.1021/ct900090f.
(11) Stoychev, G. L.; Auer, A. A.; Neese, F. Automatic Generation of Auxiliary Basis Sets. *J. Chem. Theory Comput.* **2017**, *13* (2), 554–562. https://doi.org/10.1021/acs.jctc.6b01041.
(12) Najibi, A.; Goerigk, L. The Nonlocal Kernel in van Der Waals Density Functionals as an Additive Correction: An Extensive Analysis with Special Emphasis on the B97M-V and ΩB97M-V Approaches. *J. Chem. Theory Comput.* **2018**, *14* (11), 5725–5738. https://doi.org/10.1021/acs.jctc.8b00842.
(13) Kollmar, C.; Sivalingam, K.; Helmich-Paris, B.; Angeli, C.; Neese, F. A Perturbation-based Super-CI Approach for the Orbital Optimization of a CASSCF Wave Function. *J. Comput. Chem.* **2019**, *40* (14), 1463–1470. https://doi.org/10.1002/jcc.25801.
(14) Malmqvist, P.-Å.; Roos, B. O. The CASSCF State Interaction Method. *Chemical Physics Letters* **1989**, *155* (2), 189–194. https://doi.org/10.1016/0009-2614(89)85347-3.
(15) Angeli, C.; Borini, S.; Cestari, M.; Cimiraglia, R. A Quasidegenerate Formulation of the Second Order N-Electron Valence State Perturbation Theory Approach. *The Journal of Chemical Physics* **2004**, *121* (9), 4043–4049. https://doi.org/10.1063/1.1778711.
(16) Angeli, C.; Cimiraglia, R.; Evangelisti, S.; Leininger, T.; Malrieu, J.-P. Introduction of *n* -Electron Valence States for Multireference Perturbation Theory. *The Journal of Chemical Physics* **2001**, *114* (23), 10252–10264. https://doi.org/10.1063/1.1361246.
(17) Wada, A.; Watanabe, M.; Yamanoi, Y.; Nishihara, H. Modification of the Luminescence Spectra of Chloro(Tetrapyridylcyclotetramine)Europium Complexes by Fine Tuning of the Eu–Cl Distance with Outer-Sphere Counterions in the Solid State, in a Polymer Matrix and in Solution. *Chem. Commun.* **2008**, No. 14, 1671–1673. https://doi.org/10.1039/B716987F.
(18) APEX3 Version 2016.1, Madison, Wisconsin, USA. *Bruker AXS Inc.* 2016.
(19) SAINT Version 8.37A; Bruker AXS Inc., 2015.
(20) G M Sheldrick. SADABS, 2014.
(21) Sheldrick, G. M. Crystal Structure Refinement with *SHELXL*, Version 2014/5. *Acta Crystallogr C Struct Chem* **2015**, *71* (1), 3–8. https://doi.org/10.1107/S2053229614024218.
(22) Sheldrick, G. M. A Short History of *SHELX*. *Acta Crystallogr A Found Crystallogr* **2008**, *64* (1), 112–122. https://doi.org/10.1107/S0108767307043930.

Table S1: Crystal data and structure refinement for **[Eu(L^{py})(H_2O)](PF_6)$_3$·2H_2O**.

Empirical formula	$C_{32}H_{43}EuF_{18}N_8O_3P_3$
Molecular weight MW	1174.61
Temperature/K	293.3
Crystal system	Monoclinic
Space group	C2/c
a/Å	18.0344(16)
b/Å, β/°	17.3415(13), 101.619(4)
c/Å	13.4582(19)
Volume (Å^3)	4122.7(8)
Z	4
ρ_{calc} (g/cm^3)	1.892
μ (mm^{-1})	1.766
θ range	2.09°-27.48°
R_{int}	0.0579
Measured reflections	22199
Independent reflections / unique (I >2σ (I)	4734 / 4213
Goodness-of-fit on F^2	1.078
R_1	0.0650
wR_2 (all data)	0.1839
Larg. diff. peak and hole (eÅ^{-3})	2.35 and -0.94

Table S2: Crystal structure refinement agreement factors obtained for correct ([Eu(L^{py})(H_2O)](PF_6)$_3$·2H_2O) and incorrect atom assignments.

Empirical formula	**[Eu(L^{py})(H_2O)](PF_6)$_3$·2H_2O**	**[Eu(L^{py})Cl](PF_6)$_3$·2H_2O**	**[Eu(L^{py})F](PF_6)$_3$·2H_2O**
ρ_{calc} (g/cm^3)	1.892	1.922	1.896
Goodness-of-fit on F^2	1.078	1.084	1.082
R_1	0.0650	0.0694	0.0650
wR_2 (all data)	0.1839	0.1994	0.1833
Larg. diff. peak and hole (eÅ^{-3})	2.35 and -0.94	2.506 and -1.806	2.367 and -0.978

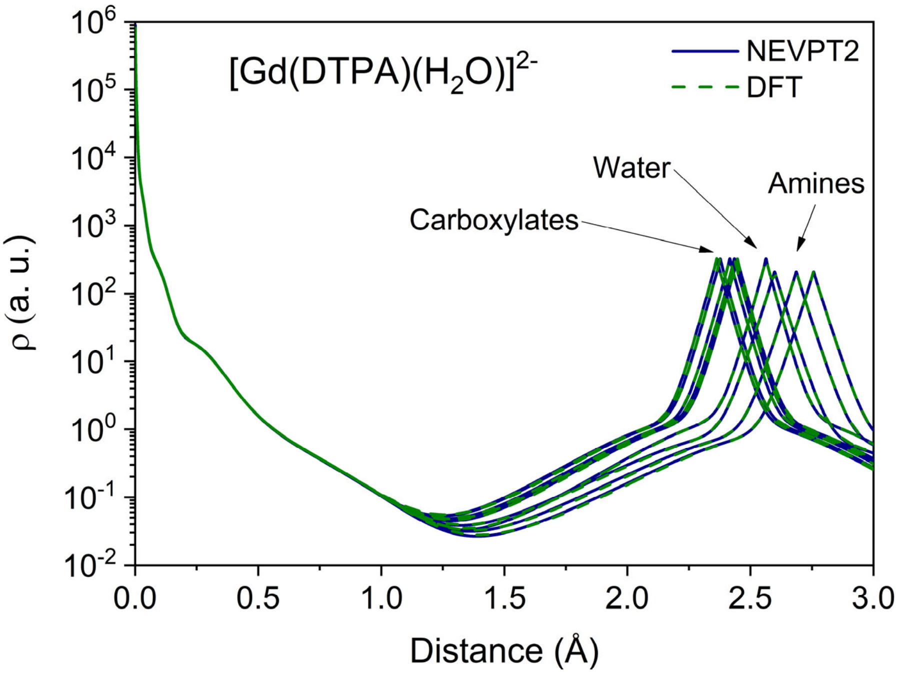

Figure S1: Electron densities (ρ) along the Gd-donor paths for [Gd(DTPA)(H$_2$O)]$^{2-}$ obtained using relativistic DFT (wB97X-D3BJ) and NEVPT2 calculations. The two methods provide nearly identical ρ values. CSD code FEPREY.

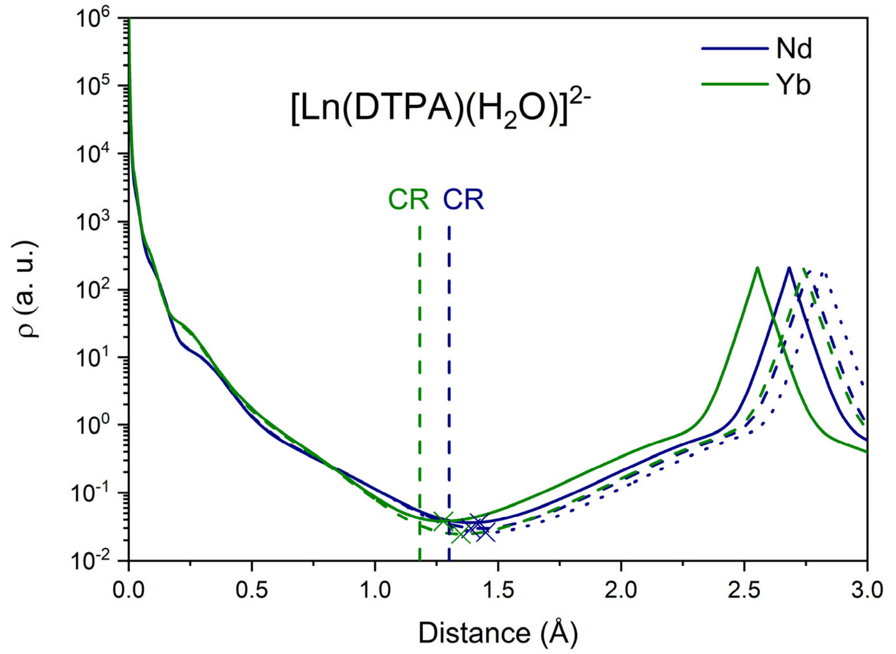

Figure S2: Electron densities (ρ) along the Ln-N$_A$ paths for [Nd(DTPA)(H$_2$O)]$^{2-}$ and [Yb(DTPA)(H$_2$O)]$^{2-}$ obtained using relativistic DFT (wB97X-D3BJ). CSD codes CUVZOI and KOLGIB, respectively. CPs are indicated with crosses.

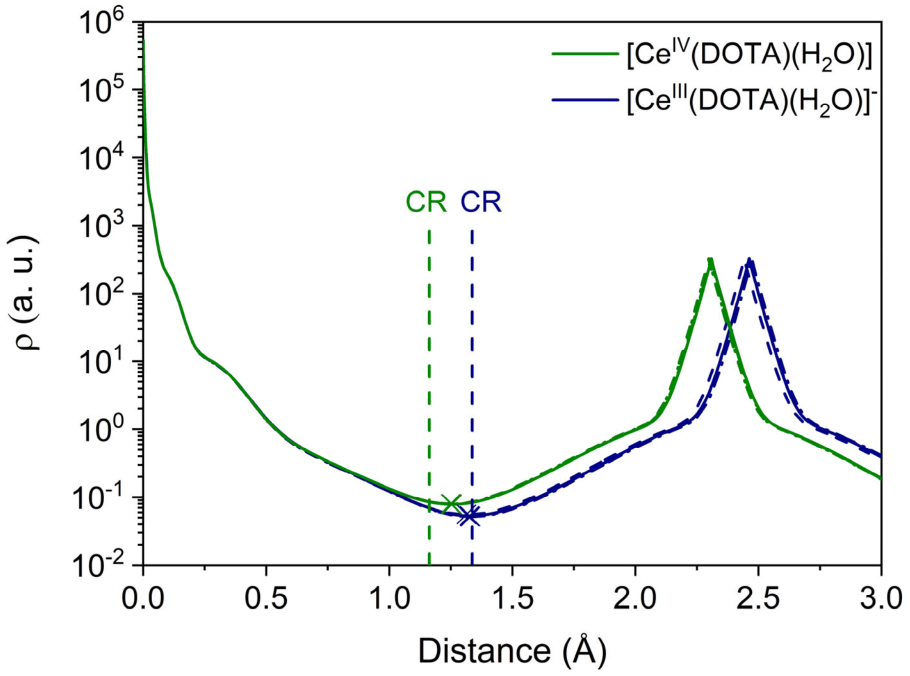

Figure S3: Electron densities (ρ) calculated along the Ce-O_C paths for $[Ce^{IV}(DOTA)(H_2O)]$ and $[Ce^{III}(DOTA)(H_2O)]^-$ using relativistic DFT (wB97X-D3BJ). CSD codes JEZREP and LUQBII, respectively. CPs are indicated with crosses.

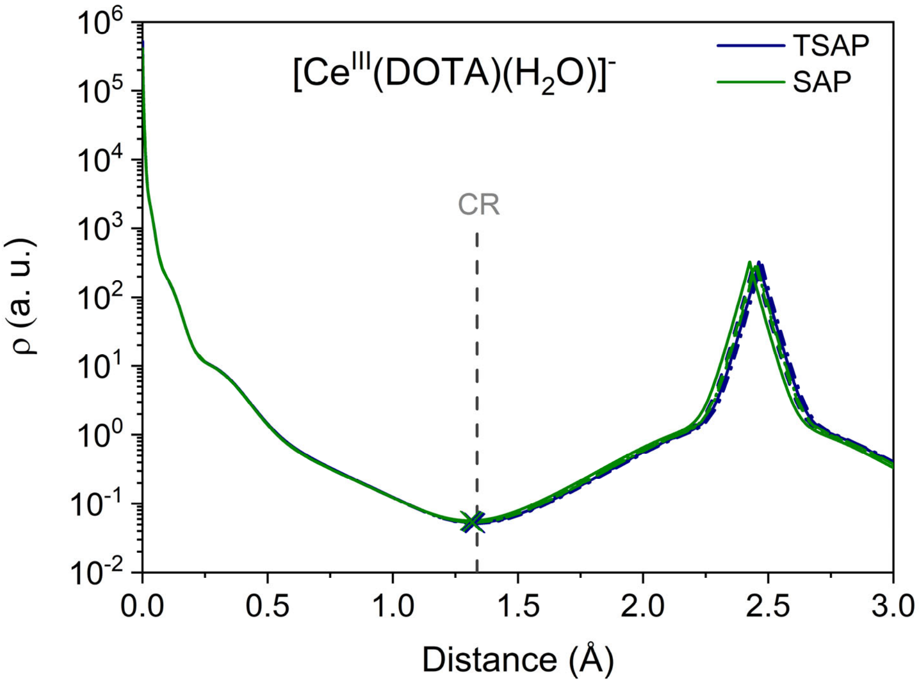

Figure S4: Electron densities (ρ) calculated along the Ce-O_C paths for the SAP and TSAP isomers of $[Ce^{III}(DOTA)(H_2O)]^-$ using relativistic DFT (wB97X-D3BJ). CSD codes ETIHIB and LUQBII, respectively. CPs are indicated with crosses.

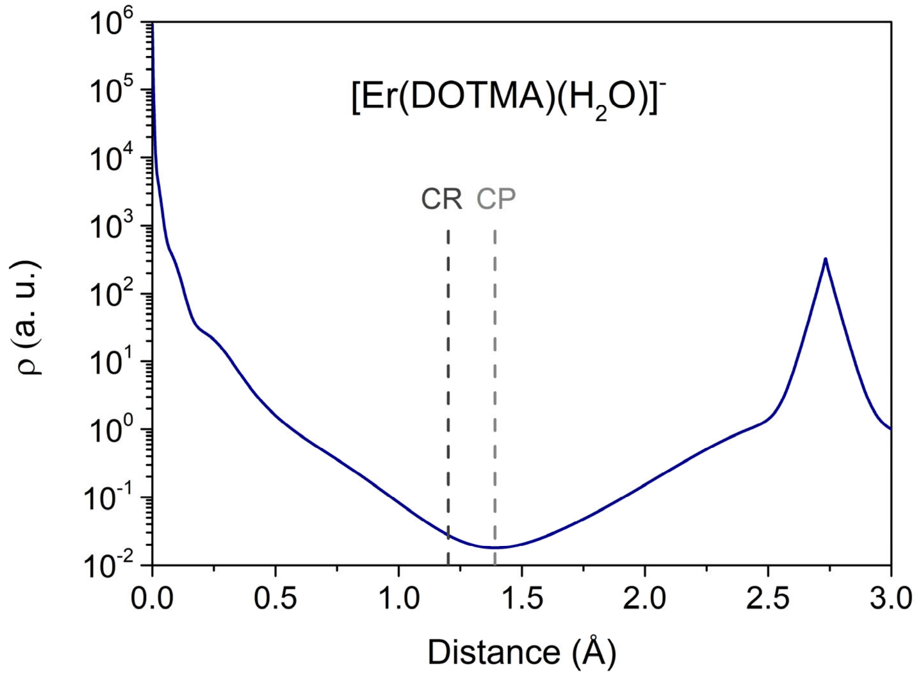

Figure S5: Electron density (ρ) calculated along the Er-O_W path for $[Er(DOTMA)(H_2O)]^-$ using relativistic DFT (wB97X-D3BJ). CSD code LOLQEL. CP indicates the position of the (3,-1) critical point, while CR denotes crystal radius.

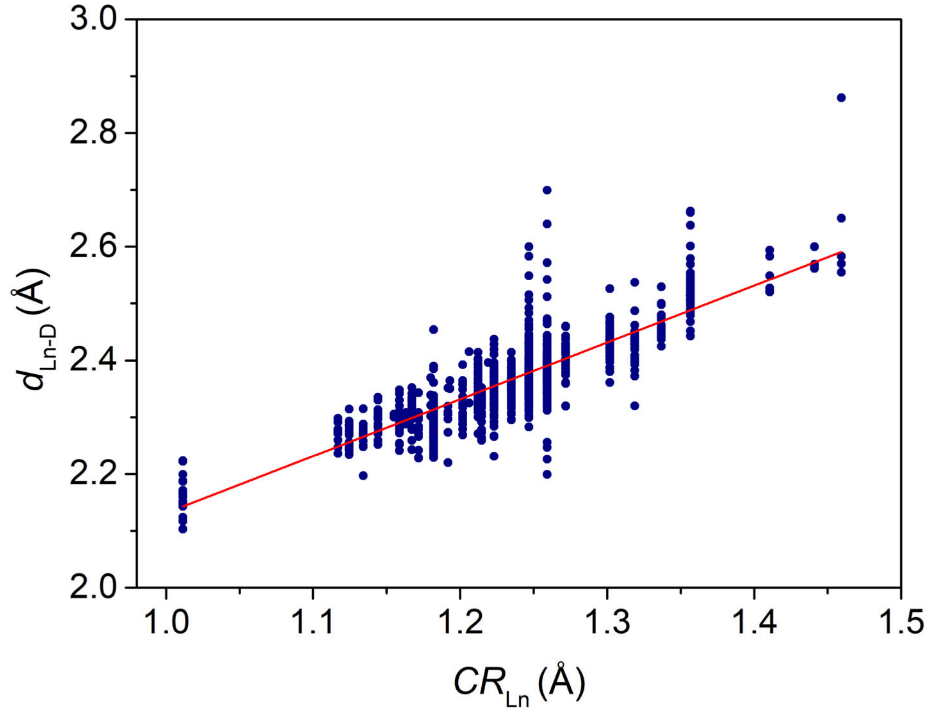

Figure S6: Plot of bond distances ($d_{Ln\text{-}D}$) versus *CR* involving carboxylate oxygen atoms (O_C).

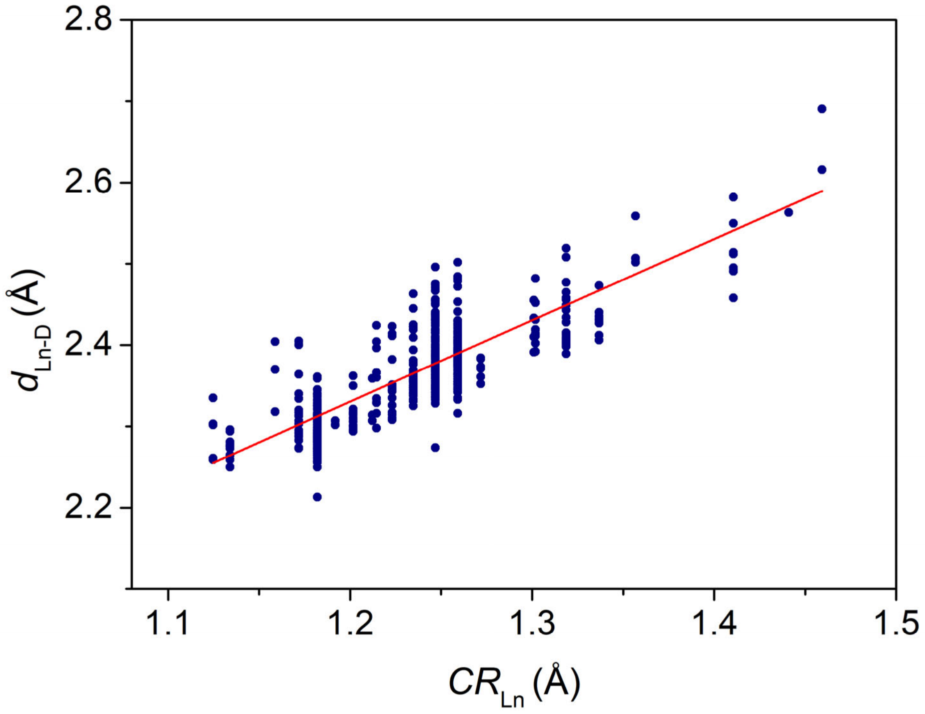

Figure S7: Plot of bond distances (d_{Ln-D}) versus *CR* involving amide oxygen atoms (O_A).

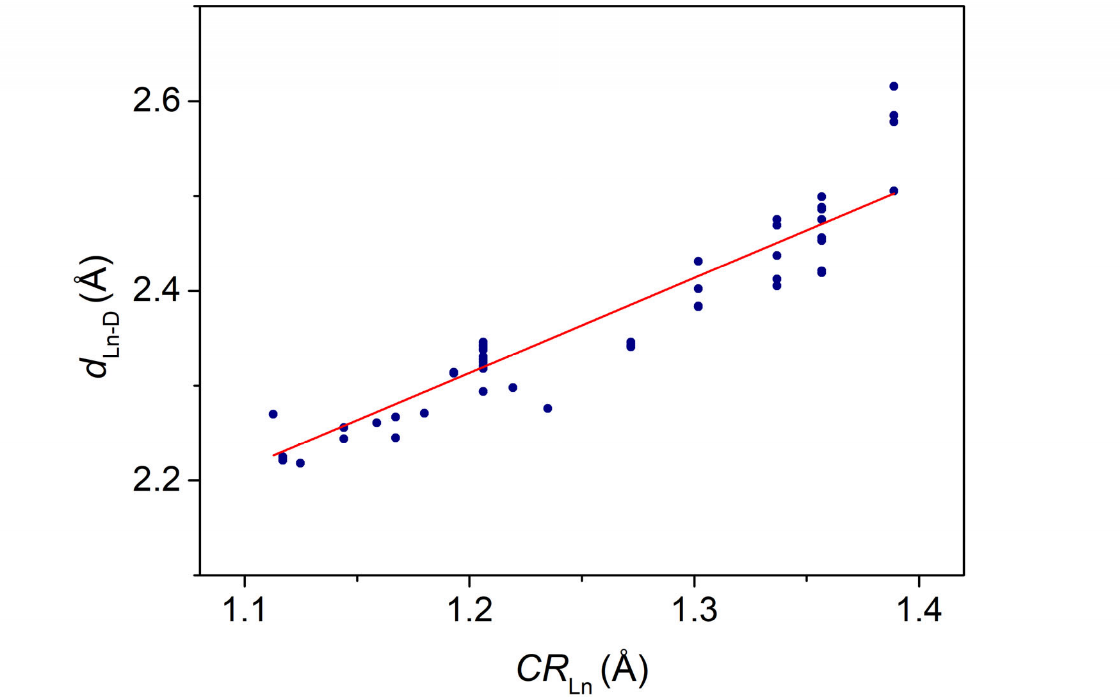

Figure S8: Plot of bond distances (d_{Ln-D}) versus *CR* involving phosphonate oxygen atoms (O_{PO3}).

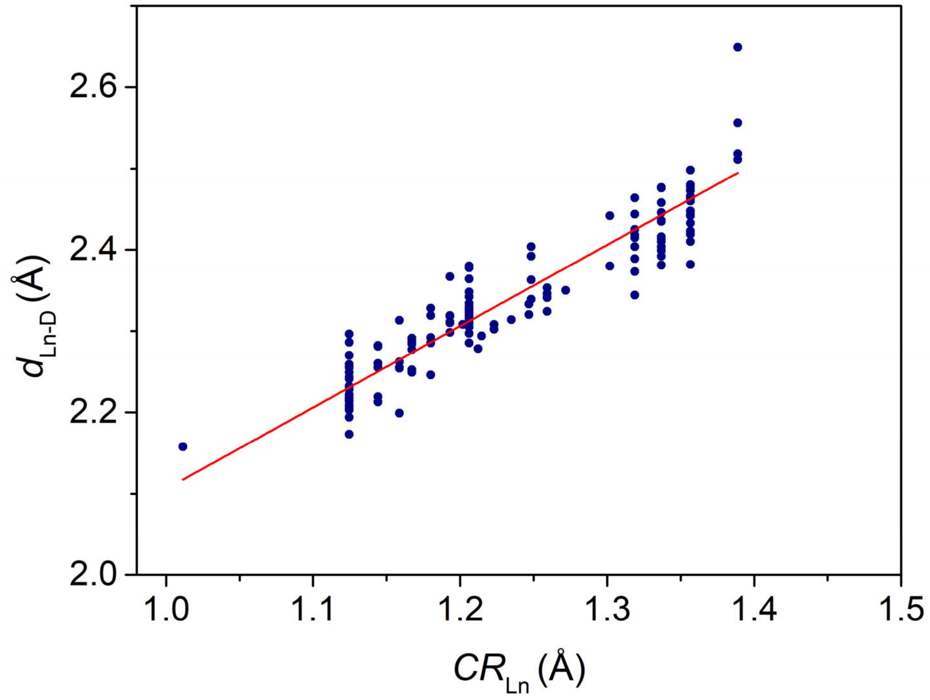

Figure S9: Plot of bond distances ($d_{Ln\text{-}D}$) versus *CR* involving phosphinate oxygen atoms (O_{PRO2}).

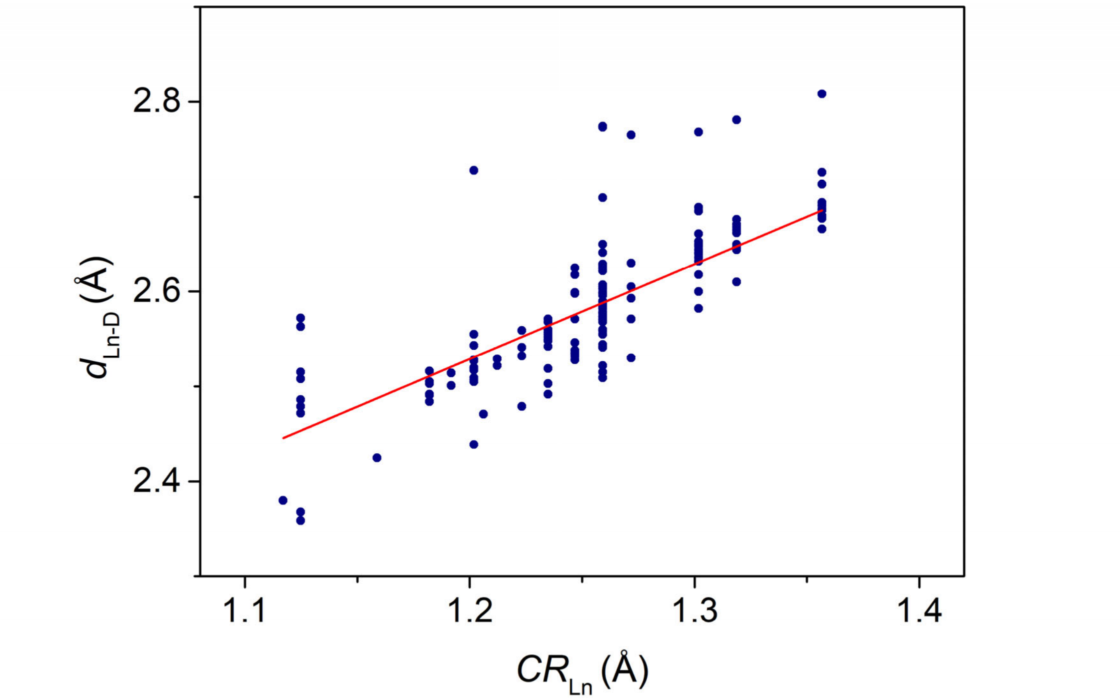

Figure S10: Plot of bond distances ($d_{Ln\text{-}D}$) versus *CR* involving pyridine nitrogen atoms (N_{PY}).

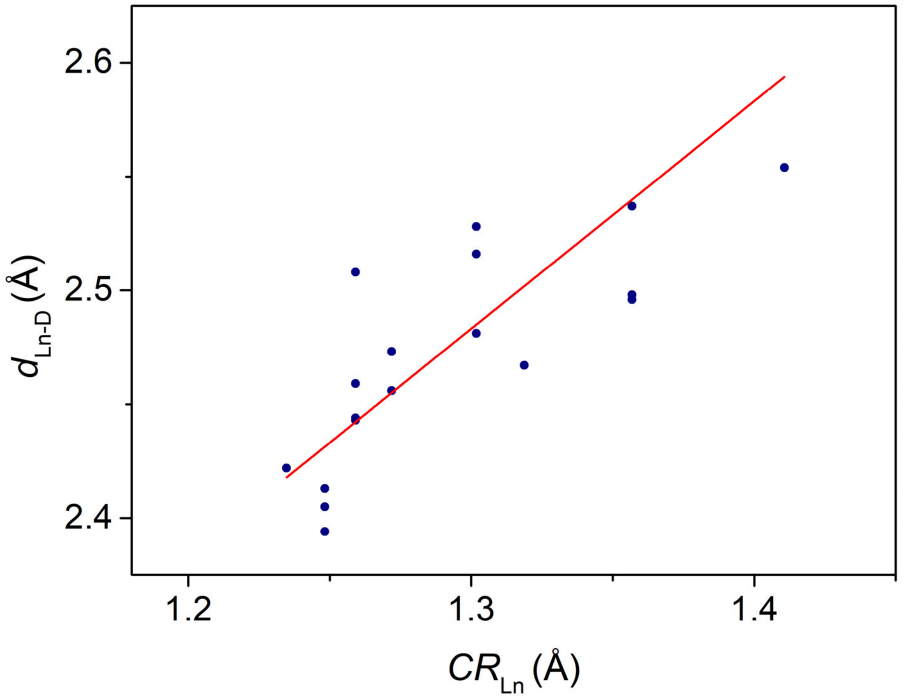

Figure S11: Plot of bond distances (d_{Ln-D}) versus *CR* involving triflate oxygen atoms(O_{Tf}).

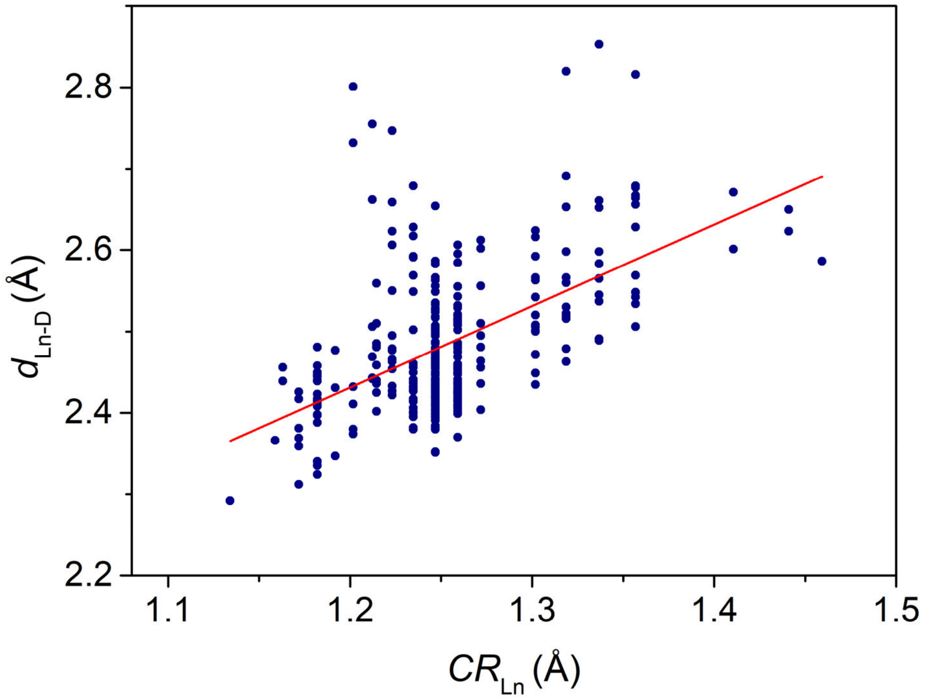

Figure S12: Plot of bond distances (d_{Ln-D}) versus *CR* involving water oxygen atoms (O_W).

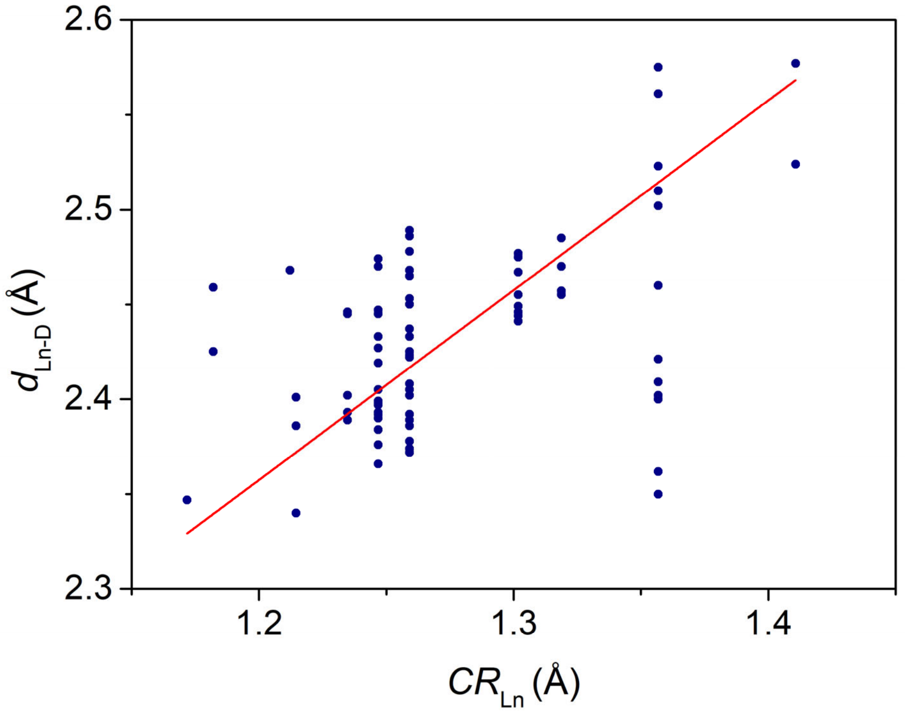

Figure S13: Plot of bond distances (d_{Ln-D}) versus *CR* involving alcohol oxygen atoms (O_{OH}).

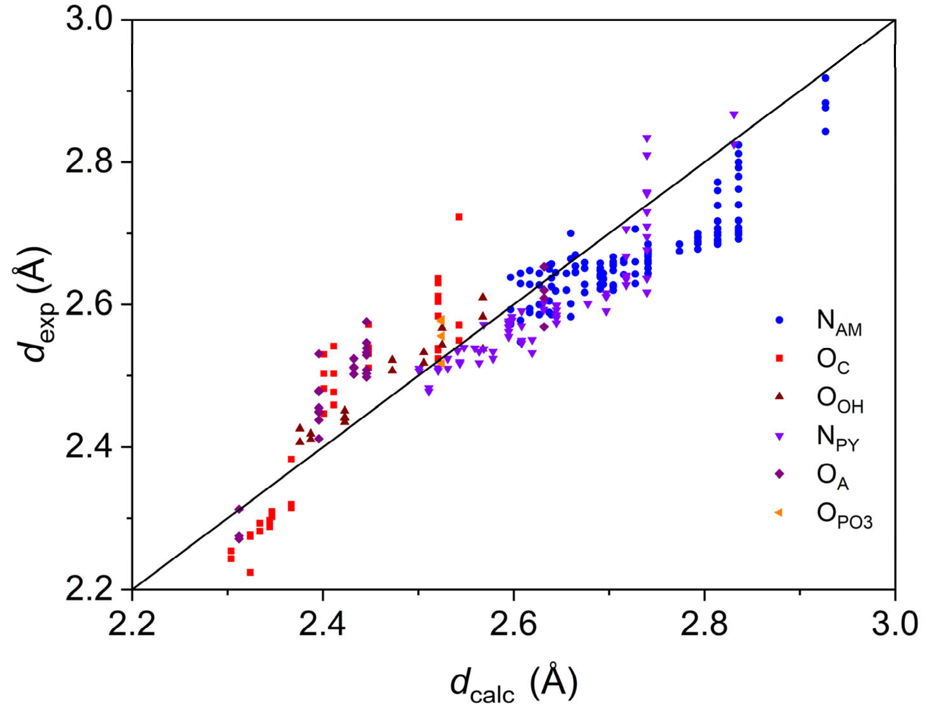

Figure S14: Plot of experimental versus calculated bond distances for H_4PYTA derivatives. The solid line corresponds to the identity function.

Table S3: Bond distances, position of the CPs, Electron density at the bond critical points (au), coordination numbers (CN), crystal radii and CSD codes.

		Ln-donor (Å)	Ln-CP (Å)	ρ_{BCP} (au)	CN	*CR* (Å)	CSD code
$[Gd(DTPA)(H_2O)]^{2-}$	Gd-N_A	2.686	1.371	0.03251	9	1.2468	FEPREY
	Gd-N_A	2.598	1.334	0.03919			
	Gd-N_A	2.756	1.399	0.02791			
	Gd-O_C	2.416	1.275	0.05006			
	Gd-O_C	2.363	1.252	0.05529			
	Gd-O_C	2.377	1.256	0.05491			
	Gd-O_C	2.446	1.289	0.04642			
	Gd-O_C	2.434	1.283	0.04821			
	Gd-O_W	2.563	1.341	0.02791			
$[Nd(DTPA)(H_2O)]^{2-}$	Nd-N_A	2.761	1.425	0.03601	9	1.3018	CUVZOI
	Nd-N_A	2.682	1.390	0.02965			
	Nd-N_A	2.822	1.450	0.02604			
	Nd-O_C	2.467	1.317	0.04833			
	Nd-O_C	2.466	1.317	0.04921			
	Nd-O_C	2.361	1.268	0.06039			
	Nd-O_C	2.443	1.307	0.05221			
	Nd-O_C	2.526	1.346	0.04410			
$[Yb(DTPA)(H_2O)]^{2-}$	Yb-N_A	2.742	1.349	0.02462	9	1.1821	KOLGIB
	Yb-N_A	2.553	1.279	0.03803			
	Yb-N_A	2.553	1.278	0.03789			
	Yb-O_C	2.295	1.188	0.05853			
	Yb-O_C	2.324	1.202	0.05392			
	Yb-O_C	2.320	1.202	0.05350			
	Yb-O_C	2.303	1.193	0.05716			
	Yb-O_C	2.291	1.185	0.05913			
$[La(DOTAM)]^{3+}$	La-O_A	2.391	1.307	0.05872	8	1.3010	PIBGOW
	La-O_A	2.433	1.327	0.05414			
	La-O_A	2.455	1.339	0.05127			
	La-O_A	2.41	1.315	0.05663			
$[La(DOTAM)(TfO)(EtOH)]^{3+}$	La-O_A	2.495	1.356	0.04635	10	1.4107	PIRSEO
	La-O_A	2.491	1.350	0.04766			
	La-O_A	2.550	1.378	0.04196			
	La-O_A	2.582	1.394	0.03901			
$[Eu^{III}(DOTP)]^{5-}$	Eu-O_{PO3}	2.325	1.237	0.06239	8	1.2061	AXAMET
	Eu-O_{PO3}	2.325	1.237	0.06238			
	Eu-O_{PO3}	2.325	1.237	0.06238			
	Eu-O_{PO3}	2.325	1.237	0.06239			
$[Eu^{II}(DOTP)]^{6-}$	Eu-O_{PO3}	2.578	1.346	0.03676	8	1.3889	ONETEJ
	Eu-O_{PO3}	2.616	1.364	0.03307			
	Eu-O_{PO3}	2.505	1.314	0.04281			
	Eu-O_{PO3}	2.585	1.350	0.03515			
$[Ce^{IV}(DOTA)(H_2O)]$	Ce-O_C	2.297	1.250	0.08013	9	1.1631	JEZREP
	Ce-O_C	2.308	1.255	0.07875			
	Ce-O_C	2.306	1.253	0.07926			
	Ce-O_C	2.299	1.250	0.07996			
$[Ce^{III}(DOTA)(H_2O)]^-$ (TSAP)	Ce-O_C	2.462	1.326	0.05332	9	1.3368	LUQBII
	Ce-O_C	2.472	1.330	0.05183			
	Ce-O_C	2.462	1.327	0.05191			
	Ce-O_C	2.442	1.314	0.05578			
$[Ce^{III}(DOTA)(H_2O)]^-$ (SAP)	Ce-O_C	2.425	1.308	0.05685	9	1.3368	ETIHIB
	Ce-O_C	2.446	1.319	0.05420			
	Ce-O_C	2.449	1.320	0.05434			
	Ce-O_C	2.452	1.321	0.05396			
$[Er(DOTMA)(H_2O)]^-$	Er-O_W	2.732	1.391	0.01790	9	1.2018	LOLQEL

Table S4: CSD codes and bond distances (Å) observed in X-ray structures of lanthanum complexes.

CODE	CN	La-N_{AM}	La-O_C	La-O_A	La-Cl	La-F	La-O_{PO3}	La-O_{PRO2}	La-N_{PY}	La-O_{Tf}	La-O_w	La-O_{OH}
PEZZUQ	9	2.713							2.666	2.496		
		2.768							2.677			
		2.705							2.687			
									2.713			
									2.808			
LESPUU	9	2.719	2.468	2.507							2.534	
		2.842	2.486	2.559								
		2.954	2.481									
WAMHIE	9	2.852	2.601								2.677	
		2.865	2.5									
		2.88	2.489									
			2.513									
			2.579									
	9	2.792	2.52									
		2.805	2.521									
		2.869	2.533									
			2.554									
			2.662									
			2.452									
	9	2.841	2.443									
		2.826	2.529									
		2.868	2.66									
			2.524									
			2.544									
			2.553									
	10	2.808	2.527								2.671	
		2.838	2.583									
		2.922	2.549									
			2.521									
			2.52									
			2.594									
WAMHOK	9	2.826	2.544									
		2.792	2.536									
		2.761	2.514									
			2.506									
			2.509									
	9	2.886	2.535									
		2.78	2.527									
		2.762	2.516									
			2.497									
			2.513									
WAMHUQ	9	2.842	2.5329									
		2.8319	2.5131									

		2.7718	2.5072									
			2.5051									
			2.5073									
	9	2.9198	2.6374									
		2.8265	2.5479									
		2.8131	2.5422									
			2.5144									
			2.5137									
YEWZAB	11	2.888	2.57	2.616							2.586	
		2.827	2.555	2.69								
		2.797	2.65									
			2.583									
			2.862									
	11	2.888	2.57	2.616							2.586	
		2.827	2.555	2.69								
		2.797	2.65									
			2.583									
			2.862									
AQILUH	10	2.989		2.512						2.554		2.577
		2.784		2.514								
		2.808		2.458								
		2.842										
CIQYOQ	9	2.863						2.442			2.656	
		2.899						2.443				
		2.898						2.478				
		2.866						2.46				
	9	2.866						2.433			2.628	
		2.883						2.448				
		2.874						2.48				
		2.843						2.442				
CUQSIR	9	2.788										
		2.76										
		2.747										
		2.827										
DUBGAL	9	2.853					2.499				2.667	
		2.831					2.419					
		2.846					2.486					
		2.841					2.453					
		2.86					2.475				2.679	
		2.813					2.488					
		2.818					2.456					
		2.837					2.421					
EKIZON	9	2.692									2.569	2.402
		2.647										2.35

		2.689										2.4
		2.706										2.362
EKOBAH	9	2.857									2.542	2.421
		2.794										2.523
		2.801										2.502
		2.787										2.51
		2.875									2.548	2.575
		2.814										2.561
		2.785										2.46
		2.785										2.409
HOYKOV	9	2.804						2.419			2.816	
		2.807						2.382				
		2.804						2.466				
		2.827						2.463				
		2.804						2.419			2.816	
		2.807						2.382				
		2.804						2.466				
		2.827						2.463				
KAWMAY	9	2.788		2.502	2.811							
		2.788		2.502								
		2.788		2.502								
		2.788		2.502								
NONJUV	9	2.788	2.49									
		2.751	2.486									
		2.781	2.483									
		2.759	2.538									
			2.509									
	9	2.783	2.479									
		2.792	2.479									
		2.797	2.507									
		2.805	2.506									
			2.569									
PIBGOW	8	2.728		2.391								
		2.712		2.433								
		2.725		2.455								
		2.71		2.41								
PIRSEO	10	2.757		2.495							2.601	2.524
		2.853		2.491								
		2.95		2.55								
		2.857		2.582								
POGHIF	9	2.761				2.227			2.694			
		2.761							2.694			
		2.787							2.68			
		2.787							2.68			

QOXSED	9	2.703							2.694	2.537		
		2.71							2.726			
		2.724							2.686			
		2.718							2.691			
QOXSIH	9	2.726								2.498		
		2.744										
		2.74										
		2.747										
RUHMIQ	9	2.69						2.498			2.664	
		2.832						2.41				
		2.835						2.423				
		2.837						2.473				
URUMUR	9	2.809									2.506	
		2.787										
		2.747										
		2.777										
	9	2.809										
		2.787										
		2.747										
		2.777										

Table S5: CSD codes and bond distances (Å) observed in X-ray structures of cerium complexes.

CODE	CN	Ce-N_{AM}	Ce-O_C	Ce-O_A	Ce-O_{PO3}	Ce-O_{PRO2}	Ce-O_W
BABFOB	9	2.753	2.5		2.412		2.545
		2.784	2.48		2.405		
		2.796					
		2.72					
CIQYUW	9	2.876				2.436	2.652
		2.834				2.416	
		2.846				2.41	
		2.875				2.446	
	9	2.864				2.414	2.583
		2.881				2.399	
		2.838				2.404	
		2.846				2.458	
ETIHIB	9	2.733	2.425				2.537
		2.709	2.446				
		2.752	2.449				
		2.719	2.452				
HOYKUB	9	2.803				2.392	2.853
		2.79				2.476	
		2.851				2.435	
		2.769				2.381	

	9	2.803				2.392	2.853
		2.79				2.476	
		2.851				2.435	
		2.769				2.381	
LOLWOB	9	2.719	2.454				2.565
		2.726	2.437				
		2.765	2.477				
		2.74	2.437				
	9	2.719	2.454				2.565
		2.726	2.437				
		2.765	2.477				
		2.74	2.437				
LUQBII	9	2.69	2.462				2.598
		2.766	2.472				
		2.744	2.462				
		2.73	2.442				
UFIRUW	9	2.767		2.44			2.491
		2.763		2.473			
		2.77		2.412			
		2.78		2.473			
	9	2.756		2.406			2.489
		2.733		2.431			
		2.776		2.427			
		2.772		2.435			
ZUDXEE	9	2.762	2.498			2.477	
		2.779	2.439				
		2.763	2.458				
		2.795	2.529				
	9	2.762	2.498			2.477	
		2.779	2.439				
		2.763	2.458				
		2.795	2.529				
JEZREP	9	2.705	2.297				2.456
		2.672	2.308				
		2.685	2.306				
		2.686	2.299				
	9	2.678	2.294				2.439
		2.638	2.288				
		2.678	2.288				
		2.638	2.294				
JEZYIA	8	2.707			2.27		

		2.707			2.27		
		2.707			2.27		
		2.707			2.27		
JEZYOG	9	2.854			2.469		2.661
		2.82			2.475		
		2.841			2.437		
		2.84			2.469		

Table S6: CSD codes and bond distances (Å) observed in X-ray structures of praseodymium complexes.

CODE	CN	Pr-N$_A$	Pr-O$_C$	Pr-O$_A$	Pr-Cl	Pr-F	Pr-O$_{PO3}$	Pr-N$_{PY}$	Pr-O$_{Tf}$	Pr-O$_W$	Pr-O$_{OH}$
PADROE	9	2.798	2.372	2.465						2.519	
		2.683	2.409	2.398							
		2.76	2.32								
PADTEW	9	2.697	2.421	2.519							
		2.825	2.403	2.508							
		2.737	2.423								
PEZZEA	9	2.748						2.644	2.467		
		2.668						2.65			
		2.683						2.61			
								2.647			
								2.781			
EQOZAL	9	2.738		2.403						2.516	
		2.696		2.428							
		2.708		2.415							
		2.746		2.434							
IKIBAF	9	2.745								2.56	2.47
		2.682									2.457
		2.722									2.455
		2.677									2.485
KEJBEH	9	2.71		2.409						2.516	
		2.757		2.456							
		2.745		2.449							
		2.702		2.458							
LOMFUR	9	2.683	2.428							2.598	
		2.732	2.449								
		2.681	2.462								
		2.706	2.393								
	9	2.683	2.428							2.598	
		2.732	2.449								
		2.681	2.462								
		2.706	2.393								
LUQBOO	9	2.703	2.43							2.53	
		2.724	2.439								
		2.746	2.437								

		2.711	2.419								
LUQBOO01	9	2.719	2.423							2.522	
		2.708	2.438								
		2.741	2.44								
		2.705	2.427								
MOWSOJ	9	2.77	2.398							2.566	
		2.784	2.451								
		2.73	2.429								
		2.758	2.435								
	9	2.733	2.436							2.691	
		2.752	2.433								
		2.768	2.382								
		2.745	2.421								
NEWPAH	9	2.732					2.425			2.82	
		2.784					2.415				
		2.76					2.373				
		2.738					2.344				
	9	2.79					2.404			2.653	
		2.776					2.444				
		2.876					2.418				
		2.8					2.389				
	9	2.732					2.425			2.82	
		2.784					2.415				
		2.76					2.373				
		2.738					2.344				
	9	2.79					2.404			2.653	
		2.776					2.444				
		2.876					2.418				
		2.8					2.389				
UFISAD	9	2.744		2.477						2.463	
		2.751		2.413							
		2.742		2.443							
		2.766		2.401							
	9	2.723		2.413						2.479	
		2.754		2.407							
		2.73		2.389							
		2.72		2.405							
UKUHAK	9	2.681			2.765			2.676			
		2.676						2.671			
		2.708						2.668			
		2.697						2.662			

ZUDMAP	9	2.757<br>2.753<br>2.777<br>2.76	2.421<br>2.487<br>2.442<br>2.537				2.464				
	9	2.757<br>2.753<br>2.777<br>2.76	2.421<br>2.487<br>2.442<br>2.537				2.464				
VELJEF	9	2.737<br>2.758<br>2.737<br>2.758				2.192		2.65<br>2.665<br>2.665<br>2.65			

Table S7: CSD codes and bond distances (Å) observed in X-ray structures of neodymium complexes.

CODE	CN	Nd-N_A	Nd-O_C	Nd-O_A	Nd-Cl	Nd-O_{PO3}	Nd-O_{PRO2}	Nd-N_{PY}	Nd-O_{Tf}	Nd-O_{OH}	Nd-O_W
CUVZOI	9	2.761<br>2.682<br>2.822	2.467<br>2.466<br>2.361<br>2.443<br>2.526								2.616
PEZZAW	9	2.69<br>2.723<br>2.637						2.6<br>2.648<br>2.768<br>2.65<br>2.582	2.481		
QAJHEO	9	2.732<br>2.751<br>2.686	2.427<br>2.468<br>2.421<br>2.455<br>2.387								2.449
AMITEV	9	2.7<br>2.687<br>2.684<br>2.696								2.449<br>2.477<br>2.475<br>2.455	2.52
	9	2.692<br>2.692<br>2.688<br>2.679								2.441<br>2.446<br>2.444<br>2.467	2.566
BABGOC	9	2.727<br>2.768<br>2.709<br>2.761	2.451<br>2.475			2.384<br>2.383					2.542
BANXIZ	9	2.655		2.411							2.472

		2.694		2.452							
		2.702		2.431							
		2.718		2.482							
EKOBIP	9	2.731									
		2.724									
		2.762									
		2.683									
	9	2.731									
		2.724									
		2.762									
		2.683									
EKOBIP01	9	2.77									
		2.721									
		2.762									
		2.723									
	9	2.77									
		2.721									
		2.762									
		2.723									
EQOZEP	9	2.677		2.419							2.501
		2.715		2.414							
		2.69		2.402							
		2.725		2.392							
HOYLAI	8	2.733					2.404				
		2.776					2.392				
		2.725					2.339				
		2.72					2.363				
	8	2.733					2.404				
		2.776					2.392				
		2.725					2.339				
		2.72					2.363				
LANQAT	9	2.696	2.438			2.402					2.592
		2.682	2.43								
		2.771	2.431								
		2.732									
	9	2.698	2.435			2.431					2.5
		2.676	2.457								
		2.751	2.448								
		2.735									
LOLWUH	9	2.696	2.407								2.563
		2.673	2.416								
		2.703	2.463								

		2.69	2.436								
	9	2.696	2.407								2.563
		2.673	2.416								
		2.703	2.463								
		2.69	2.436								
LUQBUU	9	2.693	2.42								2.508
		2.704	2.413								
		2.689	2.426								
		2.727	2.406								
LUQBUU01	9	2.687	2.428								2.501
		2.716	2.423								
		2.7	2.402								
		2.68	2.416								
PEZYUP	9	2.668			2.747			2.641			
		2.682						2.653			
		2.668						2.641			
		2.682						2.653			
PIBBAE	9	2.683						2.644	2.528		
		2.687						2.64			
		2.672						2.685			
		2.663						2.636			
QOQFAE	9	2.692	2.413								2.506
		2.681	2.397								
		2.668	2.435								
		2.706									
SUFSOB10	8	2.6							2.405		
		2.661							2.413		
		2.612							2.394		
		2.575									
UKUGAJ	9	2.648						2.618	2.516		
		2.663						2.661			
		2.676						2.632			
		2.676						2.689			
VECKEW	9	2.685	2.431								2.435
		2.693	2.426								
		2.676	2.42								
		2.716	2.422								
ZUDLOC	9	2.749	2.388				2.442				
		2.775	2.465								
		2.749	2.427								
		2.737	2.476								
		2.749	2.388				2.442				
		2.775	2.465								
		2.749	2.427								

		2.737	2.476								
ZUDLUI	9	2.748	2.437				2.38				2.624
		2.705	2.38								
		2.732	2.441								
		2.752									

Table S8: CSD codes and bond distances (Å) observed in X-ray structures of samarium complexes.

CODE	CN	Sm-N$_{A}$	Sm-O$_{C}$	Sm-O$_{A}$	Sm-O$_{PO3}$	Sm-O$_{PRO2}$	Sm-N$_{PY}$	Sm-O$_{Tf}$	Sm-O$_{W}$
EGIBON	9	2.625	2.443						2.464
		2.64	2.417						
		2.763	2.459						
			2.359						
			2.428						
	9	2.625	2.443						2.464
		2.64	2.417						
		2.763	2.459						
			2.359						
			2.428						
HAPJAM	9	2.611	2.421						
		2.645	2.422						
		2.728	2.437						
			2.429						
			2.402						
			2.46						
	9	2.611	2.421						
		2.645	2.422						
		2.728	2.437						
			2.429						
			2.402						
			2.46						
PEZZIE	9	2.729					2.63	2.456	
		2.655					2.765		
		2.649					2.593		
							2.571		
							2.605		
BABGUI	8	2.63	2.396		2.298				
		2.631	2.396		2.298				
		2.63							
		2.631							
BABHAP	9	2.686	2.422		2.341				2.556
		2.725	2.393		2.346				
		2.705							
		2.692							

	9	2.709	2.443		2.343				2.602
		2.695	2.374		2.342				
		2.718							
		2.752							
EQOZIT	9	2.654		2.383					2.495
		2.685		2.361					
		2.697		2.373					
		2.647		2.352					
ETIJAV	9	2.697	2.388						2.481
		2.677	2.379						
		2.662	2.385						
		2.66	2.393						
LOLXAO	9	2.668	2.411						2.51
		2.661	2.361						
		2.673	2.426						
		2.687	2.396						
	9	2.668	2.411						2.51
		2.661	2.361						
		2.673	2.426						
		2.687	2.396						
OGIZAG	9	2.693	2.42						2.404
		2.724	2.403						
		2.63	2.319						
		2.757	2.414						
	9	2.693	2.42						2.404
		2.724	2.403						
		2.63	2.319						
		2.757	2.414						
VUGSAS	9	2.674	2.386				2.53		
		2.611	2.396						
		2.682	2.424						
		2.594	2.428						
XILGOO	9	2.717		2.384				2.473	2.436
		2.633		2.371					2.456
		2.584							
		2.659							
ZUDMIX	9	2.71	2.397			2.35			2.612
		2.763	2.32						
		2.669	2.428						
		2.687							

Table S9: CSD codes and bond distances (Å) observed in X-ray structures of europium complexes.

CODE	CN	Eu-N$_{A}$	Eu-O$_{C}$	Eu-O$_{A}$	Eu-Cl	Eu-F	Eu-O$_{PO3}$	Eu-O$_{PRO2}$	Eu-N$_{PY}$	Eu-O$_{Tf}$	Eu-O$_{W}$	Eu-O$_{OH}$
APEXOJ	9	2.643	2.403									
		2.621	2.408									
		2.79	2.441									
			2.381									
			2.371									
CITLUO	9	2.788	2.442									
		2.638	2.364									
		2.646	2.373									
			2.369									
			2.473									
			2.464									
	9	2.788	2.442									
		2.638	2.364									
		2.646	2.373									
			2.369									
			2.473									
			2.464									
EGIBIH	9	2.752	2.402								2.45	
		2.615	2.436									
		2.632	2.444									
			2.346									
			2.414									
	9	2.752	2.402								2.45	
		2.615	2.436									
		2.632	2.444									
			2.346									
			2.414									
HIHFIN	9	2.813	2.405								2.455	
		2.653	2.436									
		2.652	2.32									
			2.379									
			2.358									
PADRUK	9	2.759	2.366								2.453	
		2.69	2.346								2.486	
		2.636	2.34									
PEZZOK	9	2.628							2.509	2.444		
		2.708							2.624			
		2.633							2.629			
									2.774			
									2.622			
WIPZOK	9	2.593	2.385									
		2.765	2.403	2.502								

		2.637	2.413									
			2.351									
	9	2.593	2.385									
		2.765	2.403	2.502								
		2.637	2.413									
			2.351									
XAGHIY	9	2.673	2.331	2.484								2.486
		2.754	2.394	2.482								
		2.64	2.344									
XAGQIF	9	2.889	2.562								2.623	
Eu II		2.776	2.6									
		2.76	2.568									
			2.569									
			2.568									
ZEQQET	9	2.674	2.426								2.485	
		2.591	2.416									
		2.627	2.362									
			2.389									
			2.362									
	9	2.608	2.428								2.502	
		2.622	2.404									
		2.739	2.399									
			2.367									
			2.344									
ZEQQIX	9	2.562	2.384									
		2.718	2.43									
		2.663	2.411									
			2.398									
			2.411									
			2.422									
	9	2.562	2.384									
		2.718	2.43									
		2.663	2.411									
			2.398									
			2.411									
			2.422									
	9	2.641	2.397									
		2.67	2.406									
		2.722	2.426									
			2.442									
			2.393									
			2.434									

	9	2.641	2.397									
		2.67	2.406									
		2.722	2.426									
			2.442									
			2.393									
			2.434									
ABOFIG	9	2.684		2.378							2.431	
		2.647		2.393								
		2.684		2.345								
		2.709		2.401								
ABOFOM	9	2.625		2.441								2.45
		2.662		2.39								
		2.636		2.416								
		2.62		2.406								
AQILIV	9	2.677		2.394							2.449	
		2.617		2.399							2.485	
		2.67		2.378								
		2.673										
AXAMAP	8	2.706					2.338					
		2.677					2.339					
		2.679					2.321					
		2.71					2.331					
		2.696					2.343					
		2.698					2.328					
		2.721					2.342					
		2.646					2.294					
AXAMET	8	2.705					2.346					
		2.705					2.346					
		2.705					2.346					
		2.705					2.346					
		2.732					2.325					
		2.732					2.325					
		2.732					2.325					
		2.732					2.325					
BABGIW	8	2.692	2.415				2.318					
		2.63	2.415				2.318					
		2.692										
		2.63										
CEXKUL	9	2.519	2.512								2.48	
		2.649	2.428									
		2.651	2.387									
		2.899	2.247									
CEXKUL01	9	2.66	2.372								2.484	

		2.677	2.38									
		2.664	2.373									
		2.704	2.39									
CEXKUL02	9	2.664	2.375								2.469	
		2.671	2.384									
		2.661	2.377									
		2.696	2.379									
CIZVAJ	9	2.671			2.763				2.559			
		2.623							2.596			
		2.668							2.59			
		2.665							2.599			
CIZVEN	9	2.675				2.212			2.541			
		2.675							2.578			
		2.704							2.541			
		2.704							2.578			
CIZVIR	9	2.662				2.153			2.542			
		2.645							2.587			
		2.662							2.542			
		2.645							2.587			
COKNAS	9	2.675	2.383								2.475	
		2.659	2.375									
		2.658	2.415									
		2.63	2.369									
	9	2.675	2.383								2.475	
		2.659	2.375									
		2.658	2.415									
		2.63	2.369									
EGALOO	9	2.669	2.374						2.515			
		2.671	2.38									
		2.595	2.404									
		2.585	2.414									
EQOZOZ	9	2.689		2.361							2.482	
		2.643		2.377								
		2.655		2.355								
		2.68		2.379								
FIBFEB	9	2.643		2.391							2.414	
		2.625		2.391								
		2.658		2.382								
		2.651		2.453								
FUXMIV	9	2.635	2.325								2.543	
		2.766	2.403									
		2.671	2.396									
		2.689	2.394									
	9	2.745	2.358								2.41	

		2.635	2.387									
		2.671	2.377									
		2.688	2.411									
	9	2.635	2.325								2.543	
		2.766	2.403									
		2.671	2.396									
		2.689	2.394									
	9	2.745	2.358								2.41	
		2.635	2.387									
		2.671	2.377									
		2.688	2.411									
HOYLEM	8	2.664						2.342				
		2.653						2.334				
		2.716						2.364				
		2.675						2.285				
	8	2.664						2.342				
		2.653						2.334				
		2.716						2.364				
		2.675						2.285				
HUNPOV	9	2.713	2.358	2.333								2.468
		2.637		2.393								
		2.592		2.379								
		2.723										
ISAZEH	9	2.702	2.354						2.773			
		2.683	2.343						2.522			
		2.666	2.331									
		2.682										
KAWLUR	9	2.673		2.41					2.555			
		2.731		2.41					2.555			
		2.673										
		2.731										
	9	2.716		2.421					2.544			
		2.73		2.421					2.544			
		2.716										
		2.73										
LERRIK	8	2.678						2.328				
		2.64						2.31				
		2.659						2.38				
		2.577						2.315				
	8	2.678						2.328				
		2.64						2.31				

		2.659						2.38				
		2.577						2.315				
LERROQ	8	2.677						2.325				
		2.651						2.297				
		2.661						2.378				
		2.586						2.319				
	8	2.677						2.325				
		2.651						2.297				
		2.661						2.378				
		2.586						2.319				
LOLQIP	9	2.67	2.355								2.584	
		2.652	2.36									
		2.659	2.39									
		2.603	2.402									
	9	2.67	2.355								2.584	
		2.652	2.36									
		2.659	2.39									
		2.603	2.402									
LOZVAX	9	2.624		2.364							2.417	
		2.625		2.377							2.42	
		2.647		2.342								
		2.605										
LUDRIO	9	2.661	2.322									
		2.651	2.322									
		2.651	2.388									
		2.661										
	9	2.661	2.322									
		2.651	2.322									
		2.651	2.388									
		2.661										
MACTAO	8	2.626										
		2.7										
		2.684										
		2.679										
	8	2.681										
		2.685										
		2.635										
		2.657										
MACTES	8	2.658										
		2.693										
		2.658										
		2.682										

MACTES01	8	2.659										
		2.693										
		2.683										
		2.658										
MOVXIH	9	2.641	2.358	2.417							2.458	
		2.684	2.357									
		2.636	2.336									
		2.641										
MUBGAR	9	2.661		2.369						2.444		
		2.661		2.369								
		2.661		2.369								
		2.661		2.369								
NAHDIK	9	2.685		2.383							2.486	
		2.643		2.403								
		2.676		2.386								
		2.745										
NUCFID	9	2.667	2.366						2.605			
		2.668	2.365									
		2.653	2.348									
		2.635										
NUHMOS	9	2.697	2.391								2.448	
		2.681	2.393									
		2.664	2.354									
		2.667	2.412									
OFAYEC	9	2.624	2.416					2.324			2.555	
		2.648	2.391									
		2.725	2.397									
		2.633										
	9	2.624	2.416					2.324			2.555	
		2.648	2.391									
		2.725	2.397									
		2.633										
OGIZEK	9	2.709	2.392									
		2.685	2.312									
		2.613	2.41									
		2.752	2.406									
			2.389									
	9	2.709	2.392									
		2.685	2.312									
		2.613	2.41									
		2.752	2.406									
			2.389									
OKAHAK	9	2.683	2.37						2.65			
		2.693	2.364						2.589			
		2.717	2.352									

		2.708								
ONETAF	8	2.869					2.518			
		2.819					2.649			
		2.889					2.511			
		2.861					2.556			
ONETEJ	8	2.712				2.578				
		2.798				2.616				
		2.775				2.505				
		2.74				2.585				
OVIRAP	9	2.64	2.387							2.543
		2.647	2.376							
		2.702	2.4							
		2.635	2.366							
		2.689	2.408							2.529
		2.657	2.377							
		2.654	2.386							
		2.696	2.364							
PEKDIV	9	2.796		2.563						2.65
		2.796		2.563						
		2.796		2.563						
		2.796		2.563						
PEKDOB	9	2.666		2.351						2.532
		2.652		2.379						
		2.666		2.351						
		2.652		2.379						
PIBBEI01	9	2.653			2.725			2.626		
		2.628						2.596		
		2.653						2.626		
		2.628						2.596		
PIBBEI	9	2.653			2.725			2.626		
		2.628						2.596		
		2.653						2.626		
		2.628						2.596		
PIBBOS	9	2.64						2.598	2.508	
		2.645						2.628		
		2.643						2.59		
		2.618						2.598		
PIBBUY	9	2.676						2.56		
		2.675						2.574		
		2.649						2.584		
		2.648						2.603		
	9	2.66						2.584		
		2.67						2.607		
		2.63						2.56		

		2.632							2.573			
	9	2.642							2.568			
		2.64							2.59			
		2.644							2.573			
		2.641							2.57			
PIMWUD	9	2.71									2.502	2.433
		2.72										2.378
		2.698										2.453
		2.691										2.478
	9	2.697									2.512	2.392
		2.654										2.437
		2.673										2.389
		2.706										2.425
RUHMEM	8	2.675						2.305				
		2.653						2.322				
		2.724						2.348				
		2.697						2.329				
RUVJOI	9	2.712									2.45	2.422
		2.613										2.423
		2.676										2.372
		2.671										2.386
	9	2.628									2.457	2.465
		2.674										2.402
		2.709										2.405
		2.639										2.374
SICVAE	9	2.695	2.379								2.423	
		2.729	2.382									
		2.697	2.373									
		2.691	2.389									
	9	2.709	2.38								2.425	
		2.708	2.376									
		2.716	2.373									
		2.719	2.226									
	9	2.689	2.377								2.415	
		2.713	2.372									
		2.707	2.368									
		2.685	2.47									
	9	2.715	2.38								2.426	
		2.721	2.374									
		2.705	2.366									

		2.707	2.256				
	9	2.705	2.372				2.42
		2.705	2.375				
		2.693	2.377				
		2.713	2.64				
	9	2.699	2.374				2.429
		2.691	2.372				
		2.704	2.372				
		2.701	2.318				
	9	2.712	2.38				2.429
		2.711	2.379				
		2.704	2.378				
		2.703	2.699				
	9	2.71	2.374				2.424
		2.712	2.38				
		2.699	2.375				
		2.692	2.199				
SICVEI	9	2.725	2.401				2.52
		2.736	2.412				
		2.735	2.411				
		2.729	2.394				
	9	2.719	2.41				2.516
		2.742	2.419				
		2.72	2.402				
		2.723	2.339				
	9	2.724	2.41				2.519
		2.723	2.402				
		2.727	2.4				
		2.739	2.35				
	9	2.728	2.406				2.508
		2.724	2.403				
		2.739	2.408				
		2.723	2.386				
	9	2.735	2.402				2.515
		2.727	2.405				
		2.732	2.408				
		2.721	2.572				

	9	2.722	2.4								2.513	
		2.733	2.412									
		2.718	2.399									
		2.724	2.4									
	9	2.727	2.401								2.514	
		2.738	2.403									
		2.741	2.392									
		2.746	2.542									
	9	2.727	2.406								2.509	
		2.721	2.407									
		2.733	2.399									
		2.725	2.405									
SICVIM	9	2.72	2.376								2.457	
		2.715	2.409									
		2.663	2.419									
		2.641	2.424									
	9	2.643	2.39								2.521	
		2.812	2.372									
		2.579	2.406									
		2.785	2.347									
	9	2.711	2.39								2.435	
		2.783	2.427									
		2.659	2.351									
		2.664	2.376									
	9	2.719	2.33								2.606	
		2.709	2.382									
		2.728	2.428									
		2.673	2.385									
TASHAZ	9	2.665	2.359						2.699			
		2.727	2.371									
		2.663	2.382									
		2.636										
TEGTEH	9	2.714				2.17			2.571			
		2.697							2.582			
		2.715							2.571			
		2.697							2.582			
TEGTEH01	9	2.705				2.16			2.57			
		2.694							2.577			
		2.705							2.57			
		2.694							2.577			
TUQTII	9	2.684		2.381							2.438	

		2.713		2.43							
		2.679		2.348							
		2.637		2.365							
TUQTII01	9	2.713		2.43							2.438
		2.679		2.348							
		2.637		2.365							
		2.684		2.381							
TUQTOO	9	2.688		2.374							2.425
		2.696		2.384							
		2.694		2.352							
		2.716		2.358							
TUQTOO01	9	2.688		2.374							2.425
		2.696		2.384							
		2.694		2.352							
		2.716		2.358							
TUXGIF	9	2.607	2.347	2.479							2.427
		2.673	2.35	2.404							
		2.629									
		2.674									
	9	2.68	2.381	2.426							
		2.675	2.336	2.429							
		2.679	2.39								
		2.658									
TUXGOL	9	2.688		2.405		2.218					
		2.699		2.404							
		2.706		2.399							
		2.715		2.472							
	9	2.72		2.404		2.232					
		2.678		2.414							
		2.725		2.407							
		2.682		2.421							
UKUHEO	9	2.649							2.59	2.508	
		2.652							2.641		
		2.636							2.582		
		2.65							2.599		
VECKIA01	9	2.661		2.392							2.406
		2.653		2.394							
		2.686		2.387							
		2.668		2.387							
VECKIA02	9	2.645		2.392							2.402
		2.665		2.383							
		2.68		2.387							
		2.656		2.394							
VECKIA	9	2.676		2.407							2.409

		2.662		2.389								
		2.686		2.376								
		2.651		2.397								
VESHOS	8	2.595	2.325						2.471			
		2.593	2.325						2.471			
		2.595										
		2.593										
WALVEL	9	2.612		2.41							2.37	
		2.69		2.409								
		2.66		2.424								
		2.675		2.416								
WUSKOM	8	2.637										
		2.647										
		2.665										
		2.625										
	8	2.64										
		2.68										
		2.672										
		2.649										
WUTNOQ	8	2.727										
		2.714										
		2.64										
		2.685										
	8	2.684										
		2.693										
		2.7										
		2.663										
XILGII	9	2.707		2.377						2.459	2.46	
		2.634		2.381							2.437	
		2.579										
		2.655										
XISLES	9	2.673		2.38						2.443		2.408
		2.677		2.373								
		2.658		2.363								
		2.657										
XIWPIC	9	2.663		2.335							2.414	
		2.603		2.316								
		2.611		2.343								
		2.619		2.346								
YOKBIL	9	2.726	2.381									
		2.719	2.37									
		2.691										
		2.715										

	9	2.697	2.409									
		2.71	2.395									
		2.73										
		2.678										
YURWOZ	8	2.653										
		2.627										
		2.653										
		2.663										
	8	2.634										
		2.653										
		2.686										
		2.689										
ZACXAC	9	2.662		2.395							2.441	
		2.654		2.373								
		2.635		2.403								
		2.655		2.396								
ZEWSAY	9	2.661	2.365	2.354								
		2.719		2.402								
		2.667		2.371								
		2.678		2.394								
	9	2.661	2.365	2.354								
		2.719		2.402								
		2.667		2.371								
		2.678		2.394								
ZUDLAO	9	2.707	2.341					2.341			2.595	
		2.73	2.398									
		2.677	2.397									
		2.696										
FEMKER	9	2.647	2.383								2.51	
		2.645	2.401									
		2.638	2.374									
		2.667	2.372									
FEMMOD	9	2.65	2.403								2.424	
		2.65	2.403									
		2.65	2.403									
		2.65	2.403									
FEMMUJ	9	2.655	2.4								2.436	
		2.655	2.4									
		2.655	2.4									
		2.655	2.4									
IVAMIE	9	2.652	2.379								2.457	
		2.615	2.372									
		2.691	2.363									
		2.661										

KEJMEV	9	2.724<br>2.713<br>2.673<br>2.748		2.391<br>2.379				2.346<br>2.353			2.46	
KEJMIZ	9	2.668<br>2.679<br>2.649<br>2.635	2.404<br>2.327<br>2.401<br>2.416								2.399	
ZULVIO	9	2.636<br>2.704<br>2.627<br>2.663		2.348<br>2.385<br>2.406<br>2.375								2.489

Table S10: CSD codes and bond distances (Å) observed in X-ray structures of gadolinium complexes.

CODE	CN	Gd-N$_A$	Gd-O$_C$	Gd-N$_A$	Gd-O$_{PO3}$	Gd-O$_{PRO2}$	Gd-N$_{PY}$	Gd-O$_W$	Gd-O$_{OH}$
CIQLIX	9	2.57<br>2.592<br>2.665	2.283<br>2.515<br>2.549	2.274<br>2.44				2.504	
FEPREY	9	2.686<br>2.756<br>2.598	2.416<br>2.363<br>2.377<br>2.446<br>2.434					2.563	
FIQMUN	9	2.668<br>2.636<br>2.697	2.375<br>2.408<br>2.344	2.409<br>2.431				2.439	
		2.633<br>2.801<br>2.683	2.431<br>2.396<br>2.341	2.419<br>2.456				2.463	
HEQBOU	9	2.713<br>2.612<br>2.669	2.371<br>2.402<br>2.47<br>2.392<br>2.429<br>2.463						
	9	2.713<br>2.612<br>2.669	2.371<br>2.402<br>2.47<br>2.392<br>2.429<br>2.463						
HEQBUA	9	2.672<br>2.682	2.403<br>2.348					2.418	

		2.781	2.341						
			2.459						
			2.378						
	9	2.787	2.388					2.423	
		2.649	2.381						
		2.646	2.37						
			2.492						
			2.341						
HEQBUA01	9	2.761	2.358					2.403	
		2.634	2.342						
		2.643	2.407						
			2.347						
			2.44						
	9	2.65	2.334					2.398	
		2.661	2.378						
		2.774	2.389						
			2.364						
			2.483						
LASZIO	9	2.762	2.38	2.405				2.412	
		2.61	2.382	2.45					
		2.916	2.38						
	9	2.762	2.38	2.405				2.412	
		2.61	2.382	2.45					
		2.916	2.38						
LASZOU	9	2.664	2.39	2.438				2.474	
		2.617	2.332	2.451					
		2.748	2.371						
NOMRAJ	9	2.855	2.367	2.41				2.415	
		2.644	2.394	2.467					
		2.852	2.346						
	9	2.855	2.367	2.41				2.415	
		2.644	2.394	2.467					
		2.852	2.346						
NUKRAN	9	2.657	2.455					2.495	
		2.754	2.494						
		2.677	2.45						
			2.475						
	9	2.657	2.455					2.495	
		2.754	2.494						
		2.677	2.45						
			2.475						

PEZBAX	9	2.83	2.373					2.44	
		2.587	2.352						
		2.622	2.433						
			2.442						
			2.351						
	9	2.83	2.373					2.44	
		2.587	2.352						
		2.622	2.433						
			2.442						
			2.351						
QEZGIM	9	2.618	2.31	2.424				2.437	
		2.782	2.346	2.496					
		2.782	2.346						
RIGBEO	9	2.699	2.402						
		2.668	2.331						
		2.609	2.43						
			2.325						
			2.583						
			2.44						
UDOMIJ	9	2.746	2.341	2.416				2.45	
		2.633	2.429	2.415					
		2.666	2.358						
		2.739	2.36	2.416				2.502	
		2.615	2.353	2.409					
		2.656	2.342						
UDOMOP	9	2.756	2.378	2.397				2.479	
		2.621	2.355	2.376					
		2.629	2.35						
VETDON	9	2.758	2.35	2.351				2.425	
		2.639	2.385	2.437					
		2.693	2.36						
WALQAB	9	2.785	2.369	2.454				2.432	
		2.616	2.356	2.397					
		2.699	2.387						
WAMHAW	9	2.638	2.43					2.448	
		2.704	2.388						
		2.635	2.387						
			2.373						
			2.379						
WAMHEA	9	2.677	2.438			2.32			
		2.649	2.411						
		2.724	2.391						
			2.426						
			2.419						

	9	2.677<br>2.649<br>2.724	2.438<br>2.411<br>2.391<br>2.426<br>2.419			2.32			
WIMJEI	9	2.754<br>2.797<br>2.625	2.367<br>2.377<br>2.388<br>2.35<br>2.422					2.473	
	9	2.743<br>2.624<br>2.774	2.375<br>2.373<br>2.404<br>2.354<br>2.369					2.486	
	9	2.593<br>2.762<br>2.761	2.375<br>2.388<br>2.416<br>2.365<br>2.336					2.48	
YIYLAT	9	2.571<br>2.8<br>2.615	2.417<br>2.366<br>2.36<br>2.341<br>2.388					2.464	
YOVFIY	9	2.75<br>2.665<br>2.599	2.383<br>2.371<br>2.368	2.455<br>2.427				2.442	
YOVFIY01	9	2.74<br>2.689<br>2.569	2.403<br>2.376<br>2.361	2.471<br>2.418				2.42	
YURCIX	9	2.728<br>2.62<br>2.651	2.463<br>2.403<br>2.413<br>2.376<br>2.39<br>2.364						
	9	2.728<br>2.62<br>2.651	2.463<br>2.403<br>2.413<br>2.376<br>2.39						

			2.364						
ZEBVEH	9	2.8	2.424					2.434	
		2.705	2.395						
		2.622	2.405						
			2.355						
			2.347						
ZIPJIR	9	2.695	2.35	2.454				2.408	
		2.642	2.36	2.439					
		2.783	2.357						
PAYZUM	9	2.631	2.368	2.355				2.4	
		2.748	2.387	2.475					
		2.665	2.365						
	9	2.661	2.378	2.432				2.422	
		2.643	2.322	2.474					
		2.737	2.362						
COKMUL	9	2.657	2.372					2.455	
		2.629	2.366						
		2.639	2.398						
		2.667	2.373						
	9	2.657	2.372						
		2.629	2.366						
		2.639	2.398						
		2.667	2.373						
EHOVAY	9	2.67		2.366				2.466	
		2.653		2.374					
		2.639		2.379					
		2.615		2.369					
EHUPUU	9	2.66	2.372				2.618		
		2.616	2.399						
		2.626	2.325						
		2.648							
EKEZAW	9	2.664		2.424				2.437	
		2.677		2.363					
		2.622		2.387					
		2.652		2.392					
EKOFUI	9	2.655		2.372				2.556	
		2.641		2.375					
		2.655		2.372					
		2.641		2.375					
EKOGAP	9	2.654		2.349				2.437	
		2.647		2.366					
		2.642		2.368					
		2.646		2.345					
EQOZUF	9	2.67		2.368				2.46	

		2.633		2.345					
		2.65		2.367					
		2.675		2.353					
GEGCIE	9	2.626		2.455				2.461	
		2.621		2.393					
		2.649		2.351					
		2.647		2.35					
HECWOD	9	2.714	2.37	2.359				2.5	
		2.664	2.367						
		2.697	2.382						
		2.689							
IYILUY	9	2.666	2.395					2.467	2.419
		2.666	2.339						
		2.616	2.385						
		2.598							
	9	2.653	2.345					2.468	2.447
		2.629	2.373						
		2.723	2.398						
		2.677							
IZULEV	9	2.631	2.385	2.436				2.427	
		2.674	2.356						
		2.659	2.321						
		2.685							
	9	2.631	2.385	2.436				2.427	
		2.674	2.356						
		2.659	2.321						
		2.685							
JOPJIH	9	2.655	2.363					2.458	
		2.679	2.363						
		2.648	2.37						
		2.662	2.362						
JOPJIH01	9	2.656	2.37					2.463	
		2.66	2.379						
		2.689	2.359						
		2.645	2.362						
JOPJIH02	9	2.694	2.366					2.472	
		2.656	2.368						
		2.655	2.375						
		2.668	2.383						
JOPJIH03	9	2.646	2.373					2.455	
		2.66	2.377						
		2.684	2.37						
		2.645	2.364						
JOPJIH04	9	2.661	2.376					2.455	

		2.647	2.369						
		2.685	2.362						
		2.651	2.367						
KEHZUT	9	2.671		2.373				2.395	
		2.646		2.374					
		2.652		2.389					
		2.638		2.383					
KEJBAD	9	2.648		2.368				2.395	
		2.663		2.388					
		2.647		2.351					
		2.671		2.362					
	9	2.629		2.366				2.474	
		2.65		2.346					
		2.643		2.333					
		2.653		2.379					
KUKGOM	9	2.719	2.36	2.392				2.428	
		2.715	2.336						
		2.627	2.378						
		2.639							
	9	2.719	2.36	2.392				2.428	
		2.715	2.336						
		2.627	2.378						
		2.639							
LATKIA	9	2.585	2.34						
		2.578	2.335						
		2.631	2.384						
		2.587							
	9	2.622	2.343						
		2.642	2.348						
		2.567	2.356						
		2.586							
	9	2.587	2.341						
		2.607	2.367						
		2.568	2.331						
		2.611							
LOQKEH	9	2.641	2.356				2.535	2.4	
		2.673	2.367						
		2.684	2.384						
		2.66							
NULQEQ	9	2.741	2.342						2.405
		2.672	2.342						
		2.664	2.428						

		2.647	2.43						
	9	2.741	2.342						2.405
		2.672	2.342						
		2.664	2.428						
		2.647	2.43						
OBEGUB	9	2.703	2.357						
		2.659	2.406						
		2.662	2.444						
		2.749							
	9	2.703	2.357						
		2.659	2.406						
		2.662	2.444						
		2.749							
OGIZIO	9	2.65	2.354					2.437	
		2.619	2.362						
		2.602	2.351						
		2.669	2.422						
	9	2.65	2.354					2.437	
		2.619	2.362						
		2.602	2.351						
		2.669	2.422						
OGIZOU	8	2.542	2.35						
		2.56	2.364						
		2.542	2.35						
		2.56	2.364						
PEHFUG	9	2.685		2.328				2.425	
		2.633		2.372					
		2.629		2.348					
		2.628		2.346					
PEHGAN	9	2.663		2.364				2.425	
		2.656		2.39					
		2.656		2.364					
		2.663		2.39					
PEYXOJ	9	2.681	2.371					2.533	
		2.673	2.369						
		2.694	2.375						
		2.682	2.345						
	9	2.698	2.346					2.586	
		2.698	2.335						
		2.677	2.341						
		2.637	2.348						
PIQPEN	9	2.641	2.332					2.352	

		2.654	2.349						
		2.672	2.375						
		2.673							
POHKUS	9	2.696	2.394					2.497	2.445
		2.622	2.334						
		2.624	2.31						
		2.72							
	9	2.638	2.344					2.507	
		2.653	2.384						2.397
		2.648	2.315						
		2.651							
RIDVEF	9	2.664	2.355					2.566	
		2.638	2.354						
			2.33						
		2.674	2.334						
RIWLOB	9	2.699	2.338				2.625		
		2.672	2.39						
		2.655	2.377						
		2.661	2.356						
SIRCAY	9	2.642		2.353				2.467	
		2.667		2.385					
		2.671		2.333					
		2.644		2.407					
SIRCEC	9	2.682		2.361				2.461	
		2.641		2.409					
		2.642		2.389					
		2.674		2.342					
SIRCIG	9	2.692		2.356				2.435	
		2.7		2.366					
		2.681		2.356					
		2.655		2.4					
SIRCOM	9	2.708		2.37				2.38	
		2.741		2.341					
		2.708		2.37					
		2.741		2.341					
SIRCUS	9	2.696		2.362				2.381	
		2.711		2.337					
		2.711		2.362					
		2.696		2.337					
SIRDON	9	2.649		2.43				2.384	
		2.652		2.346					
		2.654		2.358					
		2.677		2.356					
TUXTIS	9	2.647	2.344	2.367				2.404	
		2.664		2.376					

		2.634		2.393					
		2.619							
	9	2.638	2.357	2.364				2.398	
		2.649		2.362					
		2.664		2.434					
		2.651							
UFIRAC	9	2.689		2.364				2.383	
		2.659		2.345					
		2.689		2.364					
		2.659		2.345					
	9	2.677		2.366				2.351	
		2.653		2.349					
		2.677		2.366					
		2.653		2.349					
UFISEH	9	2.681		2.398				2.409	
		2.689		2.347					
		2.689		2.39					
		2.695		2.347					
	9	2.703		2.347				2.42	
		2.678		2.335					
		2.654		2.346					
		2.67		2.351					
UKUFOW	9	2.649					2.534	2.419	
		2.651					2.538		
		2.614					2.546		
		2.649					2.532		
	9	2.63					2.571	2.411	
		2.55					2.528		
		2.583					2.599		
		2.642					2.598		
UNACIY	9	2.685	2.365					2.457	2.399
		2.713	2.401						
		2.686	2.375						
		2.648							
	9	2.653	2.405					2.423	2.427
		2.615	2.344						
		2.675	2.367						
		2.659							
UNACOE	9	2.661	2.339					2.435	2.47
		2.67	2.39						
		2.642	2.314						

		2.624							
	9	2.738	2.365					2.43	2.433
		2.668	2.399						
		2.622	2.344						
		2.651							
URURAC	9	2.635		2.373				2.408	
		2.607		2.369					
		2.603		2.384					
		2.62		2.376					
	9	2.608		2.366				2.447	
		2.62		2.356					
		2.624		2.374					
		2.609		2.36					
	9	2.608		2.356				2.391	
		2.608		2.364					
		2.61		2.371					
		2.59		2.339					
UZUHAZ	9	2.679	2.36						
		2.638	2.35						
		2.671	2.506						
		2.663	2.433						
			2.42						
VEYQIZ	9	2.655	2.403					2.431	
		2.661	2.349						
		2.688	2.383						
		2.674	2.385						
VUSJIE	9	2.672	2.373					2.505	
		2.684	2.326						
		2.681	2.33						
		2.701	2.434						
YACTUT	9	2.686	2.369					2.5	
		2.674	2.391						
		2.659	2.358						
		2.704	2.373						
	9	2.686	2.369					2.5	
		2.674	2.391						
		2.659	2.358						
		2.704	2.373						
WATGED	9	2.697	2.392					2.526	
		2.651	2.355					2.46	
		2.574	2.337						
		2.704							

	9	2.664<br>2.618<br>2.677<br>2.586	2.375<br>2.337<br>2.312<br>2.446<br>2.6						
DIYDAT	9	2.698<br>2.721<br>2.669<br>2.688	2.334<br>2.389<br>2.386			2.333		2.583	
ZUDLES	9	2.649<br>2.666<br>2.658<br>2.701	2.317<br>2.38<br>2.374			2.333		2.654	
EQIYIM	8	2.667<br>2.667<br>2.667<br>2.667			2.314<br>2.314<br>2.314<br>2.314				
	8	2.653<br>2.653<br>2.653<br>2.653			2.313<br>2.313<br>2.313<br>2.313				
SONDUV	8	2.665<br>2.662<br>2.664<br>2.666							
	8	2.671<br>2.646<br>2.694<br>2.67							
SONFAD	8	2.635<br>2.636<br>2.635<br>2.636				2.31<br>2.319<br>2.31<br>2.319			
LERREG	8	2.565<br>2.646<br>2.625<br>2.67				2.367<br>2.318<br>2.298<br>2.311			
	8	2.565<br>2.646<br>2.625<br>2.67				2.367<br>2.318<br>2.298<br>2.311			
YAZNOG	9	2.608	2.338					2.549	2.384

		2.567	2.334						
		2.677	2.413						
		2.593							
	9	2.678	2399					2.528	2.366
		2.61	2.382						
		2.697	2.366						
		2.683							
	9	2.646	2.299					2.517	2.398
		2.66	2.349						
		2.654	2.385						
		2.704							
	9	2.729	2.303					2.535	2.393
		2.718	2.384						
		2.74	2.342						
		2.726							
YAZNUM	9	2.682	2.36					2.487	2.376
		2.678	2.349						
		2.653	2.381						
		2.601							
	9	2.628	2.356					2.518	2.39
		2.679	2.342						
		2.648	2.396						
		2.679							
	9	2.649	2.366					2.524	2.392
		2.677	2.339						
		2.681	2.382						
		2.618							
	9	2.645	2.353					2.556	2.392
		2.692	2.356						
		2.661	2.387						
		2.623							
ZULTUY	9	2.62		2.358					2.474
		2.612		2.336					
		2.69		2.37					
		2.48		2.386					

Table S11: CSD codes and bond distances (Å) observed in X-ray structures of terbium complexes.

CODE	CN	Tb-N$_{A}$	Tb-O$_{C}$	Tb-N$_{A}$	Tb-Cl	Tb-F	Tb-O$_{PO3}$	Tb-O$_{PRO2}$	Tb-N$_{PY}$	Tb-O$_{Tf}$	Tb-O$_{W}$	Tb-O$_{OH}$
EFUBUD	9	2.584	2.387									
		2.707	2.393									
		2.621	2.38									
			2.401									
			2.368									
			2.414									
	9	2.584	2.387									
		2.707	2.393									
		2.621	2.38									
			2.401									
			2.368									
			2.414									
NOMREN	9	2.832	2.296	2.378							2.401	
		2.862	2.343	2.445								
		2.608	2.4									
	9	2.832	2.296	2.378							2.401	
		2.862	2.343	2.445								
		2.608	2.4									
PADTIA	9	2.625	2.326	2.463								
		2.661	2.334	2.419								
		2.728	2.337									
ARULIK	8	2.678										
		2.644										
		2.645										
		2.659										
ASISEC	8	2.669										
		2.669										
		2.669										
		2.669										
BABHET	8	2.591	2.369				2.271					
		2.616	2.369				2.271					
		2.591										
		2.616										
BANXOF	9	2.637		2.357							2.437	
		2.61		2.422								
		2.652		2.361								
		2.645		2.365								
BOSJEA	9	2.622		2.354							2.45	
		2.622		2.358								
		2.643		2.368								
		2.657		2.362								
BOSJIE	9	2.629									2.427	

		2.653										
		2.677										
		2.613										
EFUMAT	9	2.606		2.381							2.437	
		2.625		2.357								
		2.638		2.381								
		2.595		2.34								
EKIZUT	9	2.669									2.549	2.402
		2.669										2.402
		2.669										2.402
		2.669										2.402
	9	2.611										2.433
		2.611										2.389
		2.602										2.393
		2.619										2.445
												2.446
ETIJEZ	9	2.617	2.355								2.456	
		2.654	2.358									
		2.691	2.334									
		2.647	2.344									
FUXMER	9	2.669	2.331								2.406	
		2.729	2.356									
		2.659	2.358									
		2.614	2.382									
	9	2.763	2.296								2.502	
		2.622	2.377									
		2.647	2.373									
		2.676	2.377									
HOVWAR	9	2.705		2.347						2.422	2.414	
		2.634		2.357								
		2.715		2.341								
		2.616										
HOYLIQ	8	2.656						2.319				
		2.678						2.292				
		2.641						2.328				
		2.63						2.246				
	8	2.656						2.319				
		2.678						2.292				
		2.641						2.328				
		2.63						2.246				
ICISIY	9	2.613	2.295	2.409							2.415	
		2.659	2.365									
		2.654	2.364									

		2.643										
	9	2.662	2.334	2.394							2.417	
		2.631	2.36									
		2.605	2.367									
		2.678										
IZULAR	9	2.635	2.351	2.425							2.432	
		2.671	2.308									
		2.643	2.376									
		2.675										
	9	2.635	2.351	2.425							2.432	
		2.671	2.308									
		2.643	2.376									
		2.675										
LARLIA	9	2.708	2.373				2.276				2.679	
		2.638	2.35									
		2.661	2.368									
		2.643										
LEBMUD	8	2.556										
		2.564										
		2.587										
		2.59										
LOLRAI	9	2.66	2.352								2.569	
		2.628	2.367									
		2.662	2.349									
		2.663	2.359									
	9	2.592	2.358								2.617	
		2.672	2.329									
		2.639	2.364									
		2.636	2.334									
	9	2.646	2.357								2.592	
		2.653	2.349									
		2.651	2.323									
		2.656	2.353									
	9	2.609	2.366								2.628	
		2.64	2.322									
		2.64	2.305									
		2.606	2.369									
LUNPOB	7	2.66										
		2.668										
		2.651										
		2.641										

LUNPUH	7	2.65										
		2.638										
		2.63										
		2.64										
PIBBIM	9	2.637			2.613				2.56			
		2.62							2.555			
		2.62							2.56			
		2.637							2.555			
RUZNUV	9	2.639		2.368							2.38	
		2.619		2.381								
		2.618		2.357								
		2.669		2.358								
TEXCEF	9	2.566	2.357						2.492			
		2.644	2.388									
		2.556	2.399									
		2.655	2.347									
TUSPUT	9	2.64		2.345							2.382	
		2.633		2.358								
		2.681		2.376								
		2.63		2.369								
TUXTOY	9	2.649	2.347	2.338							2.395	
		2.634		2.356								
		2.645		2.423								
		2.645										
	9	2.634	2.329	2.364							2.4	
		2.611		2.355								
		2.642		2.376								
		2.658										
UFIREG	9	2.624		2.355							2.461	
		2.664		2.333								
		2.642		2.35								
		2.669		2.331								
UKUGEN	9	2.662							2.568			
		2.632							2.519			
		2.599							2.571			
		2.597							2.552			
	9	2.604							2.56			
		2.61							2.548			
		2.6							2.557			
		2.592							2.542			
UMADIV	9	2.656		2.348							2.441	
		2.637		2.368							2.429	
		2.641		2.325								
		2.601										

VIFPAB	9	2.645	2.368								2.427
		2.686	2.386								
		2.665	2.334								
		2.655	2.371								
WEMZEW	8	2.546									
		2.546									
		2.546									
		2.546									
ZUDMOD	9	2.657	2.365					2.314			2.591
		2.676	2.371								
		2.682	2.316								
		2.71									
ZUDMUJ	8	2.617	2.302					2.285			
		2.612	2.321								
		2.623	2.339								
		2.544									
JEQSOR	9	2.723	2.348						2.503		
		2.617	2.34								
		2.654	2.396								
		2.69	2.361								
	9	2.723	2.348						2.503		
		2.617	2.34								
		2.654	2.396								
		2.69	2.361								
VELFUR	9	2.685				2.14			2.549		
		2.685							2.549		
		2.692							2.549		
		2.692							2.549		

Table S12: CSD codes and bond distances (Å) observed in X-ray structures of dysprosium complexes.

CODE	CN	Dy-N_A	Dy-O_C	Dy-O_A	Dy-F	Dy-O_{PO3}	Dy-O_{PRO2}	Dy-N_{PY}	Dy-O_W
AQAGUU	9	2.707	2.37						
		2.571	2.363						
		2.627	2.376						
			2.411						
			2.38						
			2.391						
	9	2.707	2.37						
		2.571	2.363						
		2.627	2.376						
			2.411						
			2.38						
			2.391						

QARLOL	9	2.741	2.382						2.477
		2.658	2.39						
		2.659	2.437						
			2.378						
			2.364						
ROGROU	9	2.724	2.363						
		2.649	2.392						
		2.609	2.362						
			2.36						
			2.324						
			2.428						
VINKAF	9	2.597	2.333						2.495
		2.685	2.395						
		2.558	2.324						
			2.419						
			2.363						
VOSBOU	9	2.608	2.349	2.349					2.463
		2.616	2.319	2.348					
		2.752	2.317						
ARULOQ	8	2.669							
		2.648							
		2.671							
		2.627							
BABGAO	8	2.574	2.334			2.245			
		2.574	2.334			2.245			
		2.581							
		2.581							
BANXEV	9	2.601		2.411					2.427
		2.632		2.345					
		2.638		2.351					
		2.643		2.351					
DIYCUM	9	2.679	2.314				2.308		2.623
		2.654	2.396						
		2.73	2.333						
		2.629							
	9	2.622	2.284				2.302		2.747
		2.654	2.344						
		2.65	2.349						
		2.732							
EQUBAT	9	2.62		2.344					2.454
		2.654		2.315					
		2.644		2.317					
		2.658		2.335					
GOYBOL	9	2.605		2.348					2.427
		2.654		2.31					

		2.643		2.349					
		2.64		2.414					
HIDSAR	9	2.642	2.3	2.423					2.433
		2.657	2.303						
		2.599	2.332						
		2.628							
JAKTEV	8	2.574	2.278						
		2.599	2.308						
		2.584	2.281						
		2.578							
LARKOF	8	2.557	2.313			2.267			
		2.596	2.341						
		2.578	2.323						
		2.575							
LEBNUE	8	2.573							
		2.538							
		2.547							
		2.567							
LOLQUB	8	2.569	2.297						
		2.586	2.315						
		2.601	2.32						
		2.592	2.334						
	9	2.639	2.333						2.659
		2.613	2.331						
		2.635	2.332						
		2.609	2.304						
		2.622	2.334						2.606
		2.663	2.32						
		2.644	2.348						
	9	2.642	2.342						
		2.644	2.334						2.55
		2.607	2.377						
		2.679	2.369						
		2.63	2.359						
LUQCAB	9	2.632	2.344		2.123				2.479
		2.612	2.325						
		2.61	2.329						
		2.638	2.352						
POGHAX	9	2.669						2.541	
		2.688						2.532	
		2.669						2.541	
		2.688						2.532	
QITVUN	7	2.659							

		2.662							
		2.659							
		2.623							
TUQTUU	9	2.657		2.343					2.422
		2.676		2.326					
		2.648		2.308					
		2.608		2.382					
TUQTUU01	9	2.657		2.343					2.422
		2.676		2.326					
		2.608		2.308					
		2.648		2.382					
UNEFID	9	2.667	2.322						2.466
		2.621	2.332						
		2.638	2.34						
		2.641	2.326						
UNEFID01	9	2.667	2.322						2.466
		2.621	2.332						
		2.638	2.34						
		2.641	2.326						
VOCSAK	9	2.605	2.266					2.559	
		2.655	2.267						
		2.654	2.231						
		2.632							
		2.833							
VUGRUL	9	2.644	2.333					2.479	
		2.56	2.371						
		2.546	2.337						
		2.645	2.382						
YEQDED	8	2.683					2.277		
		2.676					2.291		
		2.629					2.287		
		2.692					2.284		
YEQDED01	8	2.669					2.252		
		2.651					2.249		
		2.671					2.25		
		2.658					2.284		
YEDHUO	8	2.667	2.352						
		2.544	2.33						
		2.566	2.243						
		2.555	2.306						
	8	2.543	2.333						
		2.58	2.259						
		2.655	2.328						
		2.583	2.296						
YEDJAW	9	2.645	2.314						

		2.645	2.374						
		2.824	2.292						
		2.687	2.388						
			2.349						
	9	2.825	2.321						
		2.645	2.354						
		2.68	2.312						
		2.642	2.372						
			2.372						

Table S13: CSD codes and bond distances (Å) observed in X-ray structures of holmium complexes.

CODE	CN	Ho-N$_A$	Ho-O$_C$	Ho-O$_A$	Ho-F	Ho-O$_{PRO2}$	Ho-N$_{PY}$	Ho-O$_W$	Ho-O$_{OH}$
AQAHAB	9	2.617	2.36						
		2.564	2.375						
		2.707	2.366						
			2.368						
			2.349						
			2.402						
	9	2.617	2.36						
		2.564	2.375						
		2.707	2.366						
			2.368						
			2.349						
			2.402						
EFUSAA	9	2.691	2.363						
		2.549	2.354						
		2.601	2.353						
			2.359						
			2.339						
			2.392						
	9	2.691	2.363						
		2.549	2.354						
		2.601	2.353						
			2.359						
			2.339						
			2.392						
EZIPIP	9	2.598	2.346					2.506	
		2.552	2.339						
		2.707	2.337						
			2.381						
			2.343						

	9	2.592	2.401					2.469	
		2.55	2.361						
		2.685	2.318						
			2.394						
			2.325						
QARLUR	9	2.703	2.332						
		2.601	2.338						
		2.573	2.377						
			2.324						
			2.339						
			2.403						
YECYUD	9	2.602	2.329						
		2.745	2.3						
		2.626	2.388						
			2.336						
			2.388						
			2.414						
	9	2.602	2.329						
		2.745	2.3						
		2.626	2.388						
			2.336						
			2.388						
			2.414						
ECOJEL	9	2.595	2.318	2.307					2.468
		2.579		2.314					
		2.651		2.359					
		2.542							
GODKOZ	9	2.642	2.331					2.443	
		2.632	2.336						
		2.664	2.33						
		2.627	2.322						
GODKOZ01	9	2.626	2.332					2.442	
		2.655	2.315						
		2.613	2.327						
		2.631	2.336						
LEBPAM	8	2.569							
		2.572							
		2.547							
		2.542							
SEYJAJ	9	2.612	2.309					2.662	
		2.616	2.314						
		2.612	2.309						
		2.616	2.314						
	8	2.547	2.3						

		2.552	2.305						
		2.547	2.3						
		2.552	2.305						
ZUDLIW	9	2.573	2.315			2.278		2.755	
		2.609	2.321						
		2.638	2.271						
		2.595							
	9	2.573	2.315			2.278		2.755	
		2.609	2.321						
		2.638	2.271						
		2.595							
VELGAY	9	2.679			2.129		2.522		
		2.663					2.529		
		2.663					2.522		
		2.679					2.529		

Table S14: CSD codes and bond distances (Å) observed in X-ray structures of erbium complexes.

CODE	CN	Er-N_A	Er-O_C	Er-O_A	Er-F	Er-O_{PO3}	Er-O_{PRO2}	Er-N_{PY}	Er-O_W
AQAHIJ	9	2.559	2.364						
		2.707	2.357						
		2.612	2.349						
			2.358						
			2.336						
			2.392						
	9	2.559	2.364						
		2.707	2.357						
		2.612	2.349						
			2.358						
			2.336						
			2.392						
BABGES	8	2.58	2.33			2.244			
		2.567	2.33			2.244			
		2.58							
		2.567							
ETIQEG	9	2.605	2.304						2.411
		2.646	2.317						
		2.621	2.317						
		2.628	2.324						
HOYLOW	8	2.653					2.255		
		2.585					2.26		
		2.616					2.281		
		2.634					2.219		

	8	2.653					2.255		
		2.585					2.26		
		2.616					2.281		
		2.634					2.219		
ISAZIL	9	2.675	2.289					2.439	
		2.634	2.269					2.728	
		2.611	2.287						
		2.628							
LARKUL	8	2.551	2.283			2.256			
		2.533	2.335						
		2.553	2.309						
		2.575							
LEBNOY	8	2.527							
		2.533							
		2.561							
		2.561							
LOLQEL	8	2.596	2.302						
		2.603	2.299						
		2.596	2.302						
		2.603	2.299						
	8	2.548	2.297						
		2.546	2.288						
		2.548	2.297						
		2.546	2.288						
	8	2.544	2.3						
		2.543	2.313						
		2.544	2.3						
		2.543	2.313						
	9	2.595	2.307						2.732
		2.6	2.301						
		2.595	2.307						
		2.6	2.301						
QITVOH	7	2.623							
		2.597							
		2.651							
		2.649							
UFIRIK	9	2.605		2.31					2.432
		2.636		2.296					
		2.628		2.294					
		2.639		2.321					
UFISIL	9	2.67		2.3					2.374
		2.681		2.362					
		2.672		2.35					

		2.653		2.306					
	9	2.661		2.315					2.38
		2.638		2.301					
		2.665		2.316					
		2.654		2.317					
UKUGOX	9	2.63						2.52	
		2.574						2.555	
		2.612						2.517	
		2.578						2.543	
	9	2.605						2.528	
		2.579						2.509	
		2.593						2.505	
		2.586						2.519	
WENBEZ	8	2.538							
		2.538							
		2.538							
		2.538							
ZUDXII	8	2.555	2.273				2.282		
		2.593	2.252						
		2.516	2.298						
		2.541							
ZUDXUU	8	2.585	2.259				2.213		
		2.529	2.271						
		2.551	2.305						
		2.573							
	9	2.577	2.287				2.308		2.801
		2.585	2.279						
		2.564	2.296						
		2.676							
VELGEC	9	2.664			2.125			2.527	
		2.672						2.527	
		2.664						2.507	
		2.672						2.507	

Table S15: CSD codes and bond distances (Å) observed in X-ray structures of thulium complexes.

CODE	CN	Tm-N_A	Tm-O_C	Tm-O_A	Tm-F	Tm-N_{PY}	Tm-O_w
ETIJID	9	2.608	2.306				2.431
		2.626	2.305				
		2.645	2.309				
		2.616	2.297				
GEBQAH	8	2.537	2.271	2.25			2.292
		2.547	2.258				

		2.553					
		2.519					
LEBNAK	8	2.523					
		2.547					
		2.548					
		2.535					
LOLQOV	8	2.507	2.197				
		2.532	2.273				
		2.518	2.268				
		2.499	2.315				
	8	2.507	2.197				
		2.532	2.273				
		2.518	2.268				
		2.499	2.315				
LOWTUO	9	2.63	2.273				2.477
		2.609	2.319				
		2.621	2.22				
		2.604	2.351				
LUQCEF	8	2.532	2.269				
		2.534	2.278				
		2.519	2.282				
		2.531	2.288				
QOQFEI	8	2.566	2.277				
		2.544	2.248				
		2.552	2.25				
		2.542					
TAVSIV	9	2.606		2.302			2.347
		2.595		2.307			
		2.606		2.302			
		2.595		2.307			
RASSAJ	8	2.536		2.259			
		2.514		2.264			
		2.581		2.296			
		2.538		2.264			
	8	2.522		2.265			
		2.546		2.294			
		2.544		2.273			
		2.507		2.276			
	8	2.52		2.296			
		2.553		2.281			
		2.525		2.275			
		2.56		2.277			
VELGIG	9	2.658			2.113	2.514	

2.658	2.514
2.669	2.501
2.669	2.501

Table S16: CSD codes and bond distances (Å) observed in X-ray structures of ytterbium complexes.

CODE	CN	Yb-N$_A$	Yb-O$_C$	Yb-O$_A$	Yb-F	Yb-O$_{PO3}$	Yb-O$_{PRO2}$	Yb-N$_{PY}$	Yb-O$_W$	Yb-O$_{OH}$
AQAHEF	9	2.697	2.334							
		2.545	2.323							
		2.6	2.335							
			2.309							
			2.385							
	9	2.697	2.334							
		2.545	2.323							
		2.6	2.335							
			2.309							
			2.385							
HABMIH	9	2.603	2.337							
		2.552	2.334							
		2.705	2.331							
			2.31							
			2.39							
	9	2.603	2.337							
		2.552	2.334							
		2.705	2.331							
			2.31							
			2.39							
KOLGIB	9	2.742	2.295					2.423		
		2.553	2.324							
		2.553	2.32							
			2.303							
			2.291							
LAMFUA	9	2.68	2.266					2.481		
		2.502	2.321							
		2.598	2.272							
			2.315							
			2.338							
	9	2.68	2.266					2.481		
		2.502	2.321							
		2.598	2.272							
			2.315							
			2.338							
AGINUB	8	2.553	2.271							

		2.519	2.276							
		2.562	2.256							
		2.448								
		2.516								
BABHIX	8	2.565	2.314			2.218				
		2.56	2.314			2.218				
		2.565								
		2.56								
BANXUL	9	2.57		2.306					2.417	
		2.608		2.302						
		2.622		2.306						
		2.608		2.361						
CAYRAX	8	2.536	2.242					2.368		
		2.52	2.247					2.359		
		2.429								
		2.429								
DIDLOS	8	2.519		2.302						
		2.528		2.259						
		2.52		2.261						
		2.529		2.303						
ECOJAH	9	2.634	2.279	2.339						2.459
		2.53		2.283						
		2.553		2.276						
		2.573								
EGOWUV	9	2.588	2.274	2.337					2.458	
		2.61	2.273							
		2.651	2.242							
		2.643								
ETIJOJ	9	2.604	2.293						2.415	
		2.622	2.282							
		2.612	2.29							
		2.643	2.297							
FIBTIT	9	2.636		2.274					2.44	
		2.626		2.298						
		2.598		2.274						
		2.63		2.288						
HOYKIP	8	2.579					2.243			
		2.616					2.259			
		2.63					2.296			
		2.606					2.205			
	8	2.579					2.243			
		2.616					2.259			
		2.63					2.296			
		2.606					2.205			
HUNPAH	9	2.549	2.454	2.299						

		2.585<br>2.632<br>2.53	2.361	2.265<br>2.316						
HUNPEL	9	2.588<br>2.569<br>2.536<br>2.507<br>2.64	2.264	2.294<br>2.295<br>2.321						
HUNPIP	9	2.586<br>2.564<br>2.5634<br>2.485<br>2.642	2.271	2.293<br>2.294<br>2.325						
IPIRAA	9	2.502<br>2.603<br>2.606<br>2.612		2.287<br>2.284<br>2.291					2.324<br>2.446	
IPIREE	9	2.629<br>2.634<br>2.515<br>2.617		2.286<br>2.289<br>2.292					2.34	2.425
IPIRII	9	2.637<br>2.528<br>2.574<br>2.463<br>2.64	2.272	2.345<br>2.28<br>2.326						
IPIROO	9	2.53<br>2.557<br>2.579<br>2.482<br>2.648	2.271	2.294<br>2.332<br>2.293						
IPIRUU	9	2.647<br>2.527<br>2.552<br>2.498<br>2.605	2.25	2.305<br>2.281<br>2.328						
IPISAB	9	2.667<br>2.507<br>2.518<br>2.579<br>2.632	2.243	2.285<br>2.328<br><br>2.315						
IPISEF	9	2.502<br>2.645<br>2.634<br>2.512<br>2.58	2.235	2.309<br>2.296<br>2.34						

IPISIJ	9	2.635	2.231	2.337						
		2.644		2.314						
		2.509		2.29						
		2.51								
		2.583								
IPISOP	9	2.653	2.229	2.272						
		2.56		2.359						
		2.535		2.308						
		2.463								
		2.573								
	9	2.623	2.25	2.308						
		2.606		2.296						
		2.498		2.313						
		2.602								
		2.507								
LEBNEO	8	2.532								
		2.505								
		2.54								
		2.513								
LOMQUC	8	2.549					2.217	2.472		
		2.678					2.216			
		2.59					2.232			
		2.57								
	8	2.592					2.194	2.479		
		2.659					2.228			
		2.557					2.219			
		2.553								
LOMRAJ	8	2.524					2.173	2.515		
		2.63					2.194	2.508		
		2.532								
		2.605								
LONVAO	8	2.505	2.245							
		2.512	2.244							
		2.524	2.255							
		2.514	2.285							
	8	2.539	2.293							
		2.517	2.286							
		2.504	2.275							
		2.525	2.286							
QIGZUE	9	2.658	2.291							
		2.701	2.287							
		2.68								
		2.653								

	9	2.68<br>2.661<br>2.687<br>2.661	2.26<br>2.284							
QIHBAN	9	2.591<br>2.617<br>2.618<br>2.645	2.246<br>2.265						2.45	
	9	2.632<br>2.577<br>2.641<br>2.624	2.257<br>2.276						2.411	
QOQFIM	8	2.536<br>2.526<br>2.541<br>2.52	2.243<br>2.235<br>2.255							
RUHMOW	8	2.64<br>2.627<br>2.619<br>2.615					2.286<br>2.232<br>2.214<br>2.255			
	8	2.668<br>2.549<br>2.59<br>2.716					2.241<br>2.27<br>2.256<br>2.228			
SIRDAZ	9	2.617<br>2.631<br>2.613<br>2.616		2.261<br>2.278<br>2.273<br>2.302					2.388	
	9	2.623<br>2.617<br>2.633<br>2.611		2.292<br>2.265<br>2.289<br>2.286					2.448	
	9	2.598<br>2.596<br>2.624<br>2.676		2.288<br>2.269<br>2.256<br>2.29					2.408	
SIRDED	9	2.606<br>2.612<br>2.655<br>2.606		2.267<br>2.257<br>2.293<br>2.282					2.445	

	9	2.65		2.265					2.439	
		2.637		2.288						
		2.622		2.295						
		2.589		2.284						
SIRDIH	9	2.651		2.25					2.397	
		2.654		2.27						
		2.663		2.286						
		2.563		2.213						
UKUGIR	9	2.591						2.484	2.398	
		2.583						2.503		
		2.636						2.516		
		2.607						2.492		
WEMZUM	8	2.498								
		2.498								
		2.498								
		2.498								
XOHVEV	9	2.636		2.302					2.335	
		2.595		2.287						
		2.602		2.305						
		2.599		2.311						
YAXKEQ	8	2.514	2.243					2.486		
		2.507	2.249							
		2.515	2.244							
		2.522								
YAXNET	8	2.64					2.207	2.572		
		2.604					2.22			
		2.592					2.209			
		2.56								
	8	2.555					2.215	2.563		
		2.638					2.222			
		2.622					2.203			
		2.59								
ZUDXOO	8	2.536	2.269				2.249			
		2.503	2.246							
		2.576	2.287							
		2.54								
	8	2.536	2.269				2.249			
		2.503	2.246							
		2.576	2.287							
		2.54								
DEWVIO	8	2.546	2.234	2.335						
		2.514	2.28							
		2.493	2.245							
		2.523								

VELGOM	9	2.655			2.1			2.505		
		2.66						2.491		
		2.655						2.505		
		2.66						2.491		
KEYMOU	8	2.549	2.316		2.121					
		2.528	2.271							
		2.529	2.29							
		2.605								
	8	2.522	2.302		2.115					
		2.548	2.315							
		2.601	2.281							
		2.55								

Table S17: CSD codes and bond distances (Å) observed in X-ray structures of lutetium complexes.

CODE	CN	Lu-N_A	Lu-O_C	Lu-O_A	Lu-O_{PO3}	Lu-N_{PY}	Lu-O_W	Lu-O_{OH}
AHIPOY	9	2.553	2.266	2.4			2.312	
		2.744	2.299	2.405				
		2.601	2.228					
PUDTIR	9	2.739	2.296	2.295			2.359	
		2.526	2.308	2.317				
		2.601	2.297					
ABOFUS	9	2.553		2.303			2.369	
		2.542		2.32				
		2.572		2.274				
		2.565		2.34				
	9	2.579		2.364				2.347
		2.581		2.307				
		2.563		2.313				
		2.545		2.288				
FEHFAB	9	2.582	2.306					
		2.502	2.229					
		2.644	2.269					
		2.554						
	9	2.654	2.271					
		2.495	2.242					
		2.595	2.343					
		2.567						
	9	2.582	2.306					
		2.502	2.229					
		2.644	2.269					
		2.554						

	9	2.654	2.271					
		2.495	2.242					
		2.595	2.343					
		2.567						
IWUQEX	9	2.606		2.307			2.381	
		2.663		2.313				
		2.647		2.313				
		2.659		2.334				
LARLAS	8	2.526	2.271		2.225			
		2.538	2.277					
		2.521	2.298					
		2.558						
LARLEW	8	2.508	2.291		2.221			
		2.534	2.272					
		2.505	2.279					
		2.552						
NOJYIU	9	2.597	2.284				2.417	
		2.621	2.279					
		2.597	2.282					
		2.641	2.269					
UFIROQ	9	2.623		2.283			2.426	
		2.592		2.273				
		2.635		2.292				
		2.624		2.273				
VESHUY	8	2.517	2.236			2.38		
		2.522	2.236			2.38		
		2.517						
		2.522						
WIXBUD	8	2.516	2.26					
		2.516	2.275					
		2.506	2.247					
		2.512	2.273					

Table S18: CSD codes and bond distances (Å) observed in X-ray structures of yttrium complexes.

CODE	CN	Y-N_A	Y-O_C	Y-O_A	Y-O_{PO3}	Y-O_{PRO2}	Y-N_{PY}	Y-O_W	Y-O_{OH}
ABUPET	9	2.764	2.332	2.404					2.401
		2.655	2.302	2.424					
		2.607	2.306						
LESPOO	8	2.758	2.348	2.404				2.366	
		2.589	2.341	2.37					
			2.32						
	8	2.758	2.348	2.404				2.366	
		2.589	2.341	2.37					

			2.32						
YEFHUM	9	2.562	2.331	2.334				2.402	
		2.623	2.321	2.36					
		2.74	2.341						
XOLGIO	9	2.737	2.347					2.44	
		2.586	2.338						
		2.586	2.341						
			2.331						
			2.336						
HODCEI	8	2.435	2.282	2.318					
		2.388	2.241						
		2.437	2.254						
		2.414							
IYENAD	8	2.565	2.272				2.425		
		2.569	2.272				2.425		
		2.565							
		2.569							
KEJROH	9	2.697	2.324			2.294		2.485	
		2.663	2.332						
		2.676	2.308						
		2.712							
	8	2.62	2.289			2.262			
		2.572	2.28						
		2.534	2.304						
		2.574							
LARLOG	8	2.555	2.33		2.261				
		2.573	2.31						
		2.565	2.303						
		2.593							
LATKOG	9	2.666	2.324					2.436	
		2.636	2.317						
		2.633	2.327						
		2.648	2.324						
LATKOG01	9	2.666	2.328					2.425	
		2.633	2.319						
		2.628	2.328						
		2.655	2.329						
LIJFAL	8	2.639				2.313			
		2.671				2.255			
		2.664				2.254			
		2.671				2.199			
POHLAZ	9	2.664	2.352					2.459	2.386
		2.739	2.292						
		2.628	2.264						
		2.627							

	9	2.618	2.259					2.51	2.34
		2.658	2.344						
		2.601	2.273						
		2.583							
QEZWEX	9	2.676		2.396					
		2.645		2.334					
		2.602		2.316					
		2.585							
	9	2.613		2.298					
		2.606		2.366					
		2.633		2.329					
		2.617							
VUSJOK	9	2.633	2.324					2.559	
		2.664	2.277						
		2.691	2.322						
		2.659	2.323						
	9	2.684	2.321					2.481	
		2.644	2.278						
		2.654	2.339						
		2.678	2.325						
	8	2.561	2.294						
		2.545	2.278						
		2.599	2.252						
		2.572	2.274						

Table S19: CSD codes and bond distances (Å) observed in X-ray structures of scandium complexes.

CODE	CN	Sc-N_A	Sc-O_C	Sc-O_{PRO2}
MOMLAD	8	2.442	2.222	
		2.45	2.171	
		2.349	2.103	
			2.188	
			2.152	
UPECEY	8	2.438	2.148	
		2.488	2.167	
		2.586	2.117	
			2.166	
			2.199	
JOGZEM	8	2.41	2.223	
		2.41	2.223	
		2.41	2.223	
		2.41	2.223	

LUQCIJ	8	2.443	2.143	
		2.443	2.143	
		2.443	2.143	
		2.443	2.143	
	8	2.444	2.147	
		2.444	2.147	
		2.444	2.147	
		2.444	2.147	
	8	2.437	2.16	
		2.437	2.16	
		2.437	2.16	
		2.437	2.16	
ZUDMET	8	2.441	2.187	2.158
		2.465	2.147	
		2.535	2.124	
		2.481		

Table S20: CSD codes and bond distances (Å) observed in X-ray structures of H_4PYTA derivatives.

CODE	CN	Ln	Ln-N_{AM}	Ln-O_C	Ln-O_A	Ln-O_{PO3}	Ln-N_{PY}	Ln-O_{OH}
GAXDOC	9	Y	2.625	2.302			2.517	
			2.586	2.31			2.519	
			2.637	2.302				
			2.657					
GAXDUI	9	Tb	2.583	2.32			2.518	
			2.601	2.383			2.534	
			2.664	2.315				
			2.7					
FAFTUE	10	La	2.708				2.617	2.61
			2.708				2.617	2.538
			2.704					2.61
			2.704					2.583
FAFVOA	10	Sm	2.673				2.581	2.507
			2.673				2.581	2.507
			2.674					2.522
			2.674					2.522
FAFVEQ	10	Nd	2.685				2.601	2.533
			2.685				2.601	2.518
			2.675					2.533
			2.675					2.518
FAFVIU	10	Pr	2.7				2.591	2.544
			2.7				2.591	2.544
			2.695					2.567
			2.695					2.567

FAFVAM	10	Lu	2.645				2.54	2.407
			2.645				2.54	2.407
			2.619					2.426
			2.619					2.426
FAFVUG	10	Yb	2.62				2.539	2.411
			2.62				2.539	2.411
			2.644					2.419
			2.644					2.419
IYEMOQ	10	Y	2.649				2.574	2.451
			2.649				2.574	2.451
			2.638					2.441
			2.638					2.441
			2.628				2.562	2.435
			2.628				2.562	2.435
			2.658					2.442
			2.658					2.442
HUHLIF	10	La	2.701	2.55			2.637	
			2.698	2.571			2.636	
			2.692	2.723				
			2.718	2.572				
HUHLOL	10	Ce	2.69	2.539			2.641	
			2.687	2.536			2.639	
			2.717	2.612				
			2.701	2.604				
			2.707	2.524			2.627	
			2.691	2.637			2.635	
			2.685	2.584				
			2.697	2.63				
HUHLUR	10	Sm	2.651	2.54			2.598	
			2.677	2.511			2.599	
			2.658	2.572				
			2.659	2.531				
HUHMAY	10	Tb	2.62	2.503			2.57	
			2.655	2.459			2.591	
			2.625	2.542				
			2.627	2.477				
HUHMEC	10	Dy	2.628	2.503			2.583	
			2.637	2.447			2.578	
			2.635	2.53				
			2.644	2.482				
HUHMIG	9	Ho	2.589	2.297			2.535	
			2.655	2.297			2.536	
			2.605	2.288				
			2.65					

HUHMOM	9	Er	2.6	2.282			2.51	
			2.644	2.282			2.524	
			2.586	2.293				
			2.627					
HUHMUS	9	Tm	2.589	2.277			2.511	
			2.648	2.224			2.507	
			2.594	2.274				
			2.628					
HUHNAZ	9	Lu	2.574	2.254			2.51	
			2.638	2.243			2.506	
			2.593	2.254				
			2.572					
EKIJOA	12	La	2.843		2.62		2.825	
			2.918		2.609		2.867	
			2.876		2.653			
			2.883		2.569			
EKIJUG	10	Sm	2.667		2.539		2.585	
			2.662		2.576		2.574	
			2.644		2.508			
			2.676		2.498			
			2.658		2.547		2.581	
			2.67		2.547		2.581	
			2.658		2.503			
			2.67		2.503			
			2.685		2.529		2.592	
			2.669		2.533		2.592	
			2.669		2.529			
			2.685		2.533			
EKIKAN	9	Yb	2.644		2.271		2.483	
			2.548		2.313		2.478	
			2.629		2.275			
			2.578					
MUGGED	10	Y	2.651		2.531		2.555	
			2.642		2.449		2.568	
			2.639		2.412			
			2.641		2.479			
			2.622		2.438		2.577	
			2.648		2.438		2.577	
			2.648		2.478			
			2.622		2.478			
			2.648		2.45		2.569	
			2.648		2.45		2.569	

			2.639		2.455			
			2.639		2.455			
MUGGIH	10	Eu	2.629		2.502		2.593	
			2.643		2.502		2.593	
			2.629		2.511			
			2.643		2.511			
			2.66		2.512		2.602	
			2.706		2.512		2.602	
			2.66		2.524			
			2.706		2.524			
NUKFOO	10	La	2.812			2.579	2.695	
			2.824			2.517	2.73	
			2.78			2.577		
			2.792			2.556		
QEKGOD	10	La	2.74				2.81	
			2.762				2.756	
			2.762				2.677	
			2.8				2.71	
							2.758	
							2.834	
QELGUJ	10	Ce	2.739				2.668	
			2.739				2.743	
			2.772				2.742	
			2.76				2.706	
							2.839	
							2.748	
QEKHAQ	10	Pr	2.686				2.788	
			2.678				2.769	
			2.689				2.611	
			2.693				2.616	
							2.81	
							2.845	
QEKHEU	10	Gd	2.642				2.794	
			2.663				2.787	
			2.659				2.551	
			2.663				2.532	
							2.791	
							2.764	
QEKHIY	10	Tb	2.648				2.748	
			2.667				2.773	
			2.66				2.546	
			2.657				2.55	
							2.839	
							2.721	
QEKHOE	10	Er	2.659				2.676	

			2.617				2.765	
			2.651				2.524	
			2.628				2.534	
							2.73	
							2.825	
QEKHUK	10	Tm	2.654				2.857	
			2.646				2.758	
			2.645				2.538	
			2.67				2.572	
							2.696	
							2.775	
